# ROS-targeting heterojunction-integrated GelMA microneedles for photo-responsive antioxidative action and accelerated diabetic wound healing

**DOI:** 10.7150/thno.120879

**Published:** 2025-09-12

**Authors:** Jinlan Tang, Weijun Liu, Zhengyao Zhang, Ye Yang, Wenwen Cheng, Xiaoyu Wang, Zesheng Chen, Zijian Wang, Weikang Hu

**Affiliations:** 1 School of Materials Science and Engineering, Stem Cells and Tissue Engineering Manufacture Center, Hubei University, Wuhan 430062, China.; 2 Department of Urology, Cancer Precision Diagnosis and Treatment and Translational Medicine Hubei Engineering Research Center, Zhongnan Hospital of Wuhan University, Wuhan 430071, China.; 3 Department of Spine Surgery, Wuhan Fourth Hospital, Wuhan 430033, China.; 4 Department of Biomedical Engineering, Hubei Province Key Laboratory of Allergy and Immune Related Disease, TaiKang Medical School (School of Basic Medical Sciences), Wuhan University, Wuhan 430071, China.; 5 Department of Endocrinology, Jiangxi Provincial People's Hospital, The First Affiliated Hospital of Nanchang Medical College, Nanchang 330006, China.

**Keywords:** microneedle, ROS-scavenging, heterojunction, nanozyme, wound healing

## Abstract

**Background:** Excessive oxidative stress activation in diabetic chronic wounds causes ongoing inflammation and cell dysfunction, which greatly impairs healing. Heterojunction photocatalytic nanozymes can potentially scavenge reactive oxygen species (ROS), but their clinical application faces challenges due to low photocatalytic efficiency, poor biocompatibility, and limited stability in physiological environments.

**Methods:** APTES-COF-1@MXene heterojunction nanozymes (AC-1@MXene) were synthesized using chemical methods combined with in-situ growth techniques. Double-layered hydrogel microneedle systems (ACMGM) were fabricated through UV polymerization with tips loaded with AC-1@MXene. The photocatalytic performance of the nanozymes was assessed using physicochemical methods. Biocompatibility was confirmed through biochemical assays, and the therapeutic effectiveness of ACMGM was evaluated in diabetic mouse wound models.

**Results:** AC-1@MXene multifunctional photocatalytic nanozymes were successfully developed, exhibiting both catalase and superoxide dismutase activities. These nanozymes demonstrated significantly enhanced enzymatic activity under visible light, efficiently converting H_2_O_2_ and ·O_2_^-^ into H_2_O and O_2_, thus providing strong antioxidant protection. *In vitro* tests confirmed excellent biocompatibility, while *in vivo* studies showed that ACMGM microneedles effectively facilitated transdermal delivery of nanozymes. This significantly aided diabetic wound healing and reduced local oxidative stress. Mechanistic insights revealed that tissue regeneration and repair resulted from synergistic effects, including anti-inflammatory actions, M2 macrophage polarization, angiogenesis, and increased collagen synthesis.

**Conclusion:** The ACMGM microneedle system effectively delivers nanozymes through the skin and enhances their catalytic activity upon exposure to visible light. This precisely modulates the oxidative stress microenvironment in refractory wounds, offering an innovative therapeutic strategy for diabetic wound treatment.

## Introduction

Skin wounds in diabetic patients pose a significant challenge to healthcare systems worldwide due to their high complication risk, long healing periods, related issues, and potential for disability 1-3. The prognosis of diabetic wounds is complicated by bacterial infections, hypoxia, diabetic microangiopathy, and abnormalities in inflammatory signaling 4-6. One key factor influencing this prognosis is the significantly high level of reactive oxygen species (ROS) generated during cellular metabolism in diabetic wounds 7,8. Reactive oxygen species (ROS) interact with the hostile wound microenvironment to form a positive feedback loop that worsens skin injury 9. The molecular mechanisms behind oxidative stress caused by excessive reactive oxygen species (ROS) buildup have been partially clarified. ROS inhibit prolyl hydroxylase (PHD) activity by depleting vital cofactors like ascorbate and α-ketoglutarate, which leads to the stabilization and nuclear buildup of hypoxia-inducible factor-1α (HIF-1α). Although HIF-1α activation generally encourages angiogenesis, direct damage to cells from ROS during severe oxidative stress—including DNA oxidation and lipid peroxidation—counteracts these protective effects, ultimately causing vascular dysfunction and hampered wound healing 10. Moreover, ROS also encourages pro-inflammatory M1 macrophage polarization and decreases anti-inflammatory M2 macrophage polarization 11,12, further exacerbating the inflammatory microenvironment in diabetic wounds. Given the harmful effects of excess reactive oxygen species (ROS), regulating ROS levels is considered a promising approach to promote faster healing of diabetic wounds. However, the current ROS-scavenging biomaterials that are both highly biocompatible and responsive to stimuli remain inadequate to fulfill clinical requirements.

Covalent organic frameworks (COFs) are emerging crystalline porous materials made of organic molecules linked by covalent bonds. 13. COF-1, among the first COFs reported by Yaghi and colleagues in 2005, is constructed through boronic acid-boronate ester linkages 14. While COFs generally exhibit high crystallinity and porosity with potential applications in gas separation and catalysis 15,16, COF-1 exhibits limited hydrolytic stability due to its boronic ester bonds. Furthermore, the lack of photosensitive moieties and metal active sites in the basic COF-1 structure may restrict its inherent ability for reactive oxygen species (ROS) scavenging applications 17. Meanwhile, COF-1 can be strategically functionalized through three primary approaches: (i) introduction of antioxidant functional groups, (ii) immobilization of antioxidative nanozymes, and (iii) establishment of charge transfer systems, thereby endowing the products with desirable ROS scavenging capacity 18-20. Among these approaches, charge transfer systems excel because of their responsiveness to stimuli, high feasibility, and catalytic efficiency 21. Currently, the anti-oxidative charge transfer systems based on COF and COF-1 have rarely been reported.

Heterojunction (HJT) is a special structure formed by the interfacial contact between two or more different semiconductors (or a semiconductor with a metal/insulator) 22. Due to the differences between the materials (such as bandgap width, electron affinity, etc.), effects such as band bending, potential barriers, or quantum wells are generated to regulate the movement of electrons and holes 23. In this study, the concept of HJT is introduced for the development of a COF-1 based charge transfer system. Monolayer MXene (Ti_3_C_2_) nanosheets demonstrate significant potential in constructing high-performance HJT due to their unique 2D structure, high electrical conductivity, tunable surface chemistry, and abundant terminal functional groups (e.g., -O, -F, -OH) 24,25. In the past few years, a series of MXene/CuS, MXene/Fe_3_O_4_, and MXene/CeO_2_ composite HJTs have been reported 26-28. Heterojunction-based systems (HJTs) have demonstrated the ability to scavenge ROS under near-infrared and visible light irradiation 29,30. Previous studies have elucidated the underlying mechanisms, including favorable band structure alignment and vacancy defect-mediated catalytic activity 31. Given that both COF-1 and MXene nanosheets possess appropriate electronic properties and photocatalytic capabilities, it is reasonable to infer that their integration could create an effective anti-oxidative and photo-responsive HJT system for ROS elimination.

In previous work, our group developed a series of gelatin methacryloyl (GelMA)-based hydrogel microneedles 32-34. GelMA derived from gelatin possesses good biocompatibility, biodegradability, and photo-crosslinking properties 35. GelMA-based hydrogel microneedles can absorb wound exudates, maintain a clean and moist wound microenvironment (WME), and provide physiological cues to promote cell proliferation, migration, and differentiation 36,37. The microneedle tips enable painless skin penetration, facilitating transdermal delivery of therapeutic agents, including small molecules, peptides, proteins, and nanomaterials for precision treatment 38,39. Given these advantages, GelMA-based microneedles have been widely explored as a versatile therapeutic platform for applications such as accelerating wound healing. In this study, the APTES-COF-1@MXene (AC-1@MXene) composite HJT was successfully immobilized onto the tips of GelMA microneedles for targeted *in vivo* applications. This immobilization method significantly increased the contact area between nanozymes and injured tissues, thereby boosting catalytic efficiency while effectively decreasing excessive nanozyme use and minimizing systemic effects of toxicity.

**Figure 1:**
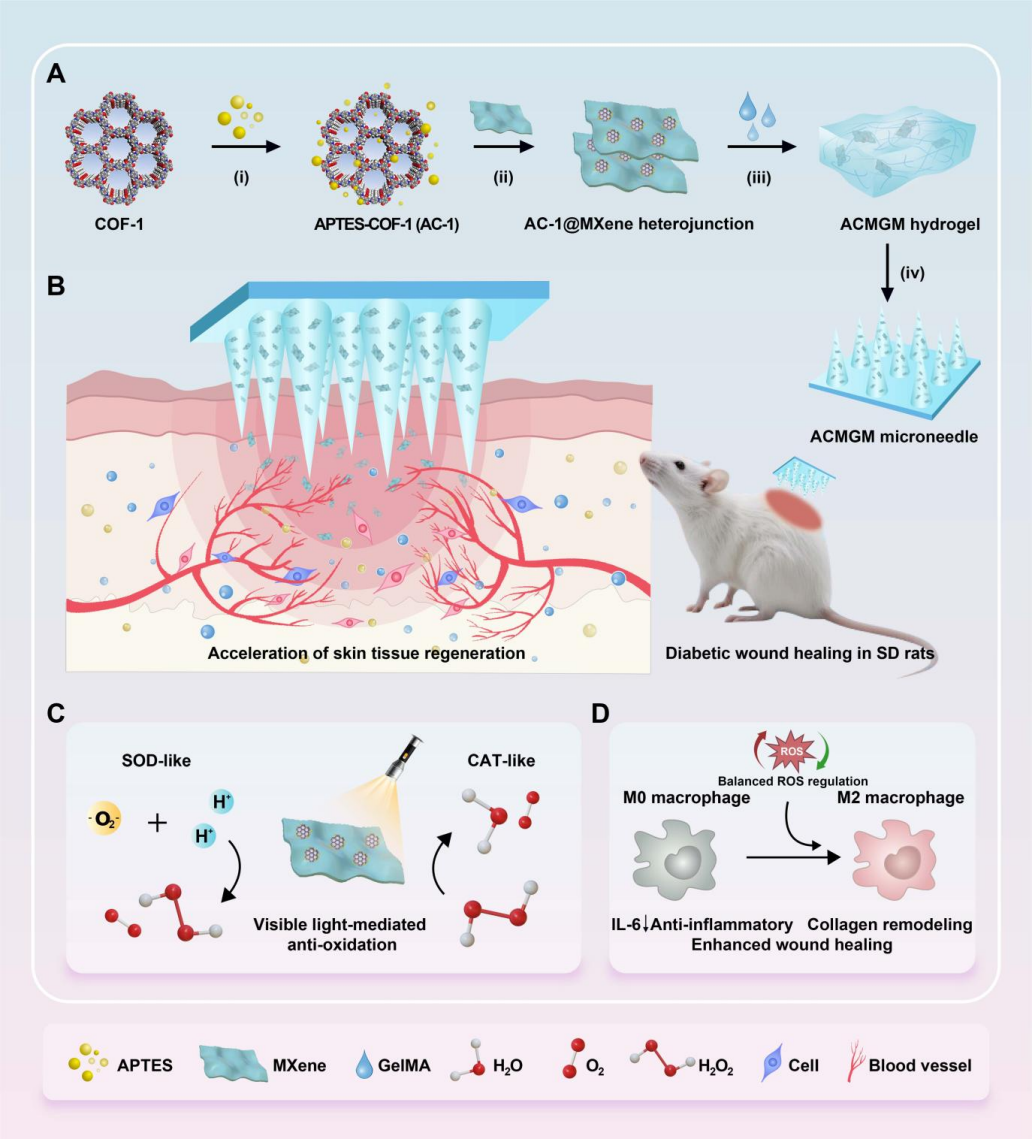
** The preparation and application of the AC-1@MXene heterojunction and the ACMGM microneedle. (A)** A diagram showing the preparation processes, including i) surface modification, ii) electrostatic self-assembly, iii) mold casting and iv) microneedle demolding; **(B)** Diabetic wound healing is accelerated by ACMGM microneedle combined with visible light therapy; **(C-D)** The potential mechanisms, including electron transfer, ROS elimination, and M2 macrophage polarization, are highlighted.

This study presents the development of an innovative anti-oxidative AC-1@MXene composite heterostructure (HJT) system integrated with a double-layer GelMA microneedle platform (**Figure 1A-B**). The core innovation lies in the architectural design of the AC-1@MXene composite, where COF-1 nanoparticles are strategically functionalized with APTES to enhance hydro-stability. This functionalization enables direct heterostructure formation with monolayer MXene nanosheets through interfacial chemical interactions, creating a previously unreported composite architecture with significantly improved functionality.

Traditional heterojunction systems, including Fe_3_O_4_/MXene and CuS@MXene composites, rely on physical mixing or surface deposition methods to load inorganic nanoparticles onto MXene nanosheets. While these conventional approaches can harness the electrical conductivity of MXene alongside the catalytic and photothermal properties of nanoparticles, they are fundamentally constrained by critical limitations: structural disorder and interface incompatibility leading to irregular distribution of active sites, limited mass transfer efficiency due to the lack of requisite porous architecture in Fe_3_O_4_ and CuS nanoparticles, and restricted surface modification capabilities that limit substrate accessibility and catalytic specificity.

Traditional nanozymes possess a single photoresponsive regulatory mechanism and lack synergistic effects, making it difficult to achieve efficient dynamic regulation of photogenerated carriers and improvement in catalytic activity. Moreover, they are limited by defects in pore structure design and insufficient microenvironment regulation capabilities, which restricts their ability to achieve efficient synergistic regulation between multiple components, thus limiting their application effects in complex wound treatment.

In contrast, the AC-1@MXene heterojunction structure is developed through an innovative in-situ growth strategy. In this approach, AC-1 is directly synthesized on MXene nanosheets to form strongly interacting, hierarchically ordered heterostructures. This design leverages the inherent advantages of both components: AC-1, as a covalent organic framework (COF), contributes its characteristic porous structure (1-5 nm) and high specific surface area, while MXene provides excellent electrical conductivity.

The amino groups (-NH_2_) and siloxane groups (Si-O-) of APTES in AC-1, along with the boroxine ring structure of the COF-1 framework, form strong interfacial coupling with the -OH/-O groups on the MXene surface through hydrogen bonding, N→B coordination interactions, and electrostatic interactions. These multifaceted interactions, where amino groups serve as N-donors that stabilize B-O bonds while establishing multiple connections with the MXene surface, create a synergistic interface that significantly enhances the separation efficiency of photogenerated charges. The heterojunction structure exposes abundant active sites and accelerates the separation and directional migration of photogenerated carriers through interfacial effects arising from work function differences and interfacial charge transfer between the two phases. This effectively suppresses the recombination probability of photogenerated electron-hole pairs, thereby achieving a significant enhancement in photocatalytic quantum efficiency.

In terms of therapeutic efficacy, the mesoporous channels within the AC-1@MXene heterojunction structure construct a biomimetic microenvironment that mimics natural enzyme active centers. The preserved porous structure of AC-1 provides efficient channels for substrate diffusion while maximizing the exposure of catalytically active sites, significantly improving mass transfer efficiency and promoting effective contact between substrates and active centers as well as rapid diffusion of products. This unique confined microenvironment, enhanced by APTES functionalization, not only improves the chemical stability of the framework but also promotes effective activation and conversion of reaction intermediates through synergistic effects at the heterostructure interface.

The system exhibits stable light-activated nanozyme activity in the wound microenvironment, precisely maintaining the dynamic balance of reactive oxygen species (ROS) through cascade enzymatic reactions and accelerating local biochemical reaction processes at the wound site. This integrated nanozyme catalytic system achieves efficient and sustained enzyme-like catalytic cycling that significantly improves the overall photocatalytic performance.

To fabricate the double-layer microneedles, AC-1@MXene HJT is immobilized onto the tips of GelMA microneedles to produce AC-1@MXene-GelMA hydrogel microneedles (ACMGM) using a mold-casting technique. The physical and chemical properties, anti-oxidative capacity, and wound healing effects of AC-1@MXene HJT and ACMGM microneedles will be thoroughly investigated. In** Figure 1C-D**, upon exposure to visible light, ACMGM microneedles are expected to generate electron-hole pairs that can produce reactive oxygen species (·O₂^-^, ·OH) for improved catalytic activity. Meanwhile, the inherent catalase (CAT)-like and superoxide dismutase (SOD)-like activities of the heterostructure allow for effective scavenging of excess ROS, thus achieving balanced oxidative regulation. This study aims to develop a biocompatible, photo-responsive microneedle system with light-activated antioxidant nanozyme for ROS elimination capabilities for diabetic wound treatment.

## Materials and Methods

### Etching of monolayer MXene nanosheets

According to our previous literature 27, Ti_3_AlC_2_ powder (1.0 g) was etched in HF/HCl mixture (15 mL each) at 35 °C for 12 h with stirring. The suspension was centrifuged (3000 rpm, 5 min) and washed three times with distilled water. The Ti_3_C_2_ precipitates were intercalated with LiCl solution (20 mL) on an orbital shaker (150 rpm, 12 h, RT), then exfoliated by ultrasonication to obtain monolayer MXene nanosheets. The products were freeze-dried for storage.

### Synthesis of AC-1@MXene nanozyme

BDBA (0.25 g) and APTES (0.075 g) were dissolved in a TMB/1,4-dioxane mixture (5 mL each). Monolayer MXene nanosheets (50 mg) were added and sonicated for 60 min. The mixture was heated to 75 °C under N₂ for 20 h to form AC-1@MXene. After cooling, the product was filtered, washed with acetone at least three times, vacuum-dried at 60 °C for 12 h, and stored in darkness.

### Characterizations of AC-1@MXene nanozyme

The morphology and element composition were characterized by scanning electron microscopy (SEM). The chemical composition and structure were further characterized by Fourier transform infrared spectroscopy (FT-IR), X-ray diffraction (XRD), and X-ray photoelectron spectroscopy (XPS). For the FT-IR test, the data within the wavenumber of 400 - 4000 cm^-1^ was recorded; For the XRD test, Cu Kα was used as a monochromatic light diffraction source to collect diffraction data in the 2θ range of 5° - 70° with a scanning rate of 10 °/min.

The values of zeta potential and particle size were measured by dynamic light scattering (DLS). Photoluminescence (PL) spectroscopy was performed using a steady-state and time-resolved photoluminescence spectrometer. For UV-Vis diffuse reflectance spectroscopy, samples were mixed with BaSO_4_ powder at a mass ratio of 1:3, ground to approximately 200 mesh, and 200 mg aliquots were analyzed using a UV-visible spectrophotometer. The ROS-generating ability was evaluated using an electron paramagnetic resonance (EPR) spectrometer.

### Photoelectrochemical properties of materials

According to a previous report 40, the photoelectrochemical properties of the AC-1@MXene nanozyme were comprehensively investigated. EIS measurements were performed in the "IMP" mode, LSV measurements were performed in the "LSV" mode, and transient photocurrent measurements were performed in the "i-t" mode. All electrode potential reported in this study was referenced to the reversible hydrogen electrode (RHE) using the conversion E(RHE) = E(SCE) + 0.241 V + 0.0591 × pH. More information about the experimental methods could be found in the Supplementary Information.

### Biochemical test of superoxide dismutase (SOD) on AC-1@MXene

100 μL each of the Met solution, NBT solution, riboflavin solution, and 1.5 mg/mL AC-1@MXene nanozyme suspension were sequentially added into 2 mL of the phosphate buffer saline (PBS), gently vortexed for 10 s, and then exposed to a Xenon light source simulating visible light (It was used in combination with an ND8 neutral density filter and a homogenizer, the output current was adjusted to 15 A, and the distance between the light source and the electrode surface was set to 15 - 30 cm, resulting in a final light intensity density of approximately 0.2 - 1.2 W/cm² at the materials surface) for 2 - 10 min. After that, 1 mL of the sample was immediately transferred into quartz cuvettes, and the absorbance values were detected using a UV-Vis spectrophotometer. At least three independent samples were detected to ensure reproducibility, and the blank control group lacking specific reagents was included to account for background absorbance.

### Enzymatic reaction kinetics assay of AC-1@MXene

An enzymatic reaction kinetics assay was performed to evaluate the enzyme catalytic capacity of the materials. The multiple enzyme catalytic efficiencies of AC-1@MXene and AC-1 were quantified with reference to the characteristic absorption spectra obtained from ultraviolet-visible (UV-Vis) spectroscopy measurements. For both superoxide dismutase SOD-like and catalase CAT-like enzyme activities, the initial concentration of nanozymes was fixed at 100 mg/mL, with a visible light irradiation power density of 0.8 W/cm² and a single irradiation duration of 8 minutes. The substrate concentration was varied accordingly, and the absorbance was measured at specific wavelengths using a UV-Vis spectrophotometer. The maximum initial reaction rate (V_m_), Michaelis constant (K_m_), and catalytic constant (K_cat_) of the enzymes were calculated by fitting the data with the Lambert-Beer law, rate equation, Michaelis-Menten equation, and Lineweaver-Burk equation.

### Flow cytometry for detecting intercellular ROS

A cell-based model of H_2_O_2_-induced oxidative stress injury was established to evaluate the anti-oxidative potential of AC-1@MXene nanozyme. The fibroblasts (L929) were seeded onto 6-well plates and cultured with complete Dulbecco's Modified Eagle Medium (DMEM) supplemented with 100 μM H_2_O_2_. Five groups were set, including blank control (B.C.), positive control (P.C.), AC-1, AC-1@MXene, and AC-1@MXene + Vis. The AC-1@MXene + Vis group was subsequently irradiated with a Xenon light source (combination with an ND8 neutral density filter and a homogenizer, 2 W/cm^2^ at the material's surface) for 10 min. After 48 h of co-incubation, the cells were collected and labeled using a DCFH-DA method. Flow cytometry was performed using a BD FACS Verse instrument with 10,000 events recorded per sample.

### Synthesis of gelatin methacryloyl (GelMA)

GelMA was chemically modified according to a previous report 27. 80 g of type A gelatin was dissolved in 720 g of distilled water at 50.0 ± 0.5 °C to prepare an aqueous gelatin solution. After that, 40 g of methacrylic anhydride (MA) was added. The mixture was reacted under constant stirring for 24 h, and dialyzed using deionized water for another 72 h. After centrifuging at 4,000 rpm for 30 min, the supernatant was collected and freeze-dried to obtain the light-yellow, foam-like GelMA.

### Photopolymerization of AC-1@MXene GelMA hydrogels

A UV-mediated photo-crosslinking strategy was used to prepare AC-1@MXene-laden GelMA hydrogels. Briefly, GelMA solution with a concentration of 15 wt% was prepared, and then mixed with AC-1@MXene nanozyme with a mass fraction of 0 %, 0.2 %, 0.5 % and 0.8 %, respectively. The mixture was stirred for 2 h for homogeneous dispersion, followed by sonication to remove air bubbles. According to a previous report 32, the photo-initiator Irgacure 2959 was added to the mixture. They were poured into a mold, and photo-cured by UV irradiation with a power of 300 W for 5 min. As a result, a series of AC-1@MXene GelMA hydrogels were prepared, and coded as AC-1@MXene-0, AC-1@MXene-0.2, AC-1@MXene-0.5 and AC-1@MXene-0.8, respectively.

### Fabrication of double-layer microneedles

A kind of microneedle (namely ACMGM microneedle) with a double-layer structure was designed for transdermal delivery of nanozyme, and it was fabricated using a mold-casting technique. The tip layer was composed of AC-1@MXene-0.5 hydrogel, and the base layer was composed of pristine GMA hydrogel. The fabrication protocols of double-layer ACMGM microneedles could be found in a previous literature 33. As the control, a single-layer GelMA microneedle (namely GM) hydrogel was also fabricated using the same protocols.

### Characterizations of composite hydrogels and microneedles

In this study, the physiochemical properties of the AC-1@MXnen GelMA hydrogels and the composite microneedles, including gelation process, morphological observation, mechanical strength and skin puncturing ability, hydrophilicity and swelling dynamics, were comprehensively characterized. The detailed experimental methods could be found in the Supplemental Information.

### Cell proliferation and migration assays

L929 cells were co-cultured with the GM and ACMGM microneedles using a trans-well chamber. For the ACMGM + Vis group, the cells in the ACMGM group were additionally irradiated with visible light for 10 min. After 24 h of incubation, the viability of treated cells was evaluated by CCK-8 assay. The value of absorbance at 450 nm was detected using a microplate reader. At least three independent samples were used for statistical analysis. For the migration assay, the L929 cells were co-cultured with the nanomaterials or microneedles for 24 h, then mitomycin C was added at a concentration of 3 μg/mL for a 2-h treatment. The cells were then seeded onto a 6-well tissue culture plate at high cell density with DMEM medium supplemented with 1% serum for migration culture. According to a previous report 41, the migration ability of cells was quantitatively evaluated using a scratching assay.

### Subcutaneous transplantation assay

This study was approved by the Animal Ethics and Welfare Committee of Hubei University (Certificate No. 20250003). It was performed in accordance with the National Institutes of Health (NIH) guidelines for the care and use of laboratory animals. Female Sprague-Dawley (SD) rats, weighing about 200 ± 10 g, were purchased from the Hubei Center for Animal Disease Control and Prevention. All animals were fed in a specific pathogen-free (SPF) environment for 7 days.

The animals were anesthetized with 3% isoflurane and randomly divided into three groups (n = 3 per group). GM and ACMGM microneedles were subcutaneously transplanted into the back skin. The control group was sham-operated. 21 days after surgery, fresh whole blood and the organs (heart, liver, spleen, lung, kidney) were harvested from each animal. According to a previous report 32, H&E staining assay and a series of blood biochemical tests were performed for biocompatibility evaluations *in vivo*.

### Diabetic wound healing assay

According to a previous report 42, a model of Type-II diabetes of SD rats was established by intraperitoneal injection of streptozotocin (STZ) as follows: Following 2 months of high-fat diet feeding, rats were subjected to 18-hour fasting before receiving intraperitoneal injection of streptozotocin (STZ) at 25 mg/kg body weight. A second STZ injection at 15 mg/kg was administered 7 days post-initial treatment based on the animals' general condition assessment. After a 2-week observation period with continuous monitoring of blood glucose, body weight, and behavioral parameters, rats with fasting blood glucose ≥11.1 mmol/L and random blood glucose ≥16.7 mmol/L were considered eligible as successfully established T2DM models. These animals were anesthetized and then randomly divided into five groups (n = 5 per group), including a control group (sham operation), GM group (GM microneedle), commercial group (3M™ Tegaderm™), ACMGM group (ACMGM microneedle), and ACMGM + Vis group (ACMGM microneedle with visible light irradiation).

Following depilation of SD rats, a full-thickness skin wound with a diameter of 15 mm was created on each animal using a sterile biopsy punch, which was immediately covered with a wound dressing (either the microneedle dressing or a commercial dressing). To further assess photothermal toxicity and determine the optimal irradiation intensity, photothermal tests were performed, with the results presented in **Figure S17**. After comprehensive evaluation, the ACMGM + Vis group received additional visible light irradiation. A Xenon lamp was used as the visible light source, with its operating current adjusted to 15 A, it was employed in combination with an ND8 neutral density filter and a homogenizer, and the optical path distance was set to 10 cm, resulting in a light intensity density of approximately 3 W/cm^2^ at the wound surface. Visible light irradiation was administered three times daily, with each exposure lasting 10 minutes, and this treatment regimen was maintained for three consecutive days. The wound area was measured at regular intervals. Neo-skin tissues at day 7 and day 14 were harvested for a series of histological analyses, such as H&E staining, Masson's staining and immunofluorescence staining (CD86, CD206, Ki67, CD31, ROS, IL-6, TNF-α, Col-I and Col-III). These studies were performed with the help of Biofavor Biotech. Co., Ltd (Wuhan, China). A laser confocal microscope was used for data acquisition.

### Statistical analysis

All data were expressed as mean ± standard deviation (SD). Statistical analysis was performed using GraphPad Prism 9.0 software. One-way analysis of variance (ANOVA) followed by Tukey's post-hoc test was employed to assess significant differences among multiple groups. Significance levels were set as follows: *n.s.* indicates no significance, *P < 0.05, **P < 0.01, ***P < 0.001.

## Results and Discussion

### Synthesis and characterization of AC-1@MXene heterojunction

**Figure 2:**
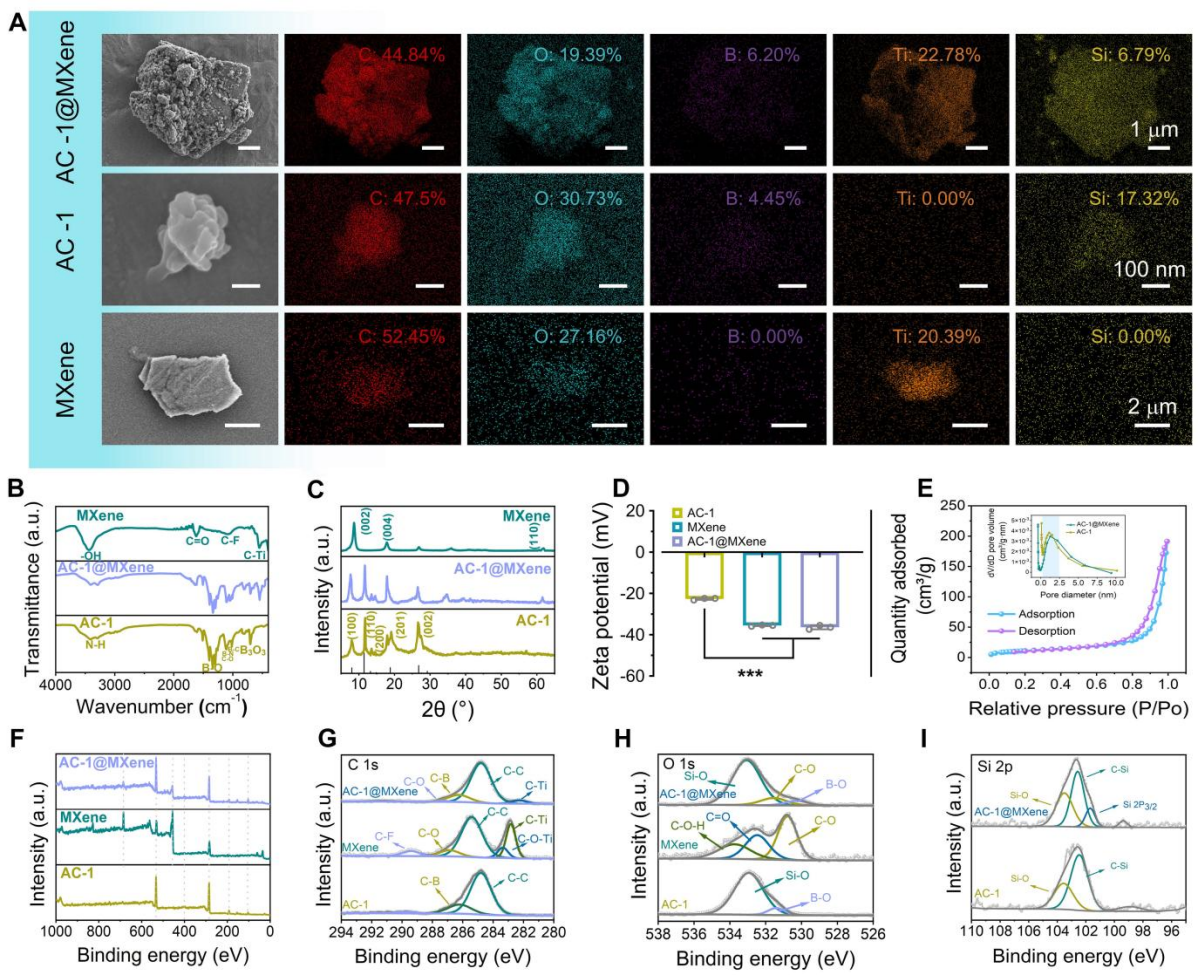
** The preparation and characterization of anti-oxidative AC-1@MXene nanozyme. (A)** SEM images of **AC-1@MXene** nanozyme, AC-1 nanoparticles and monolayer MXene nanosheets, scale bars were marked; **(B)** FT-IR spectrum; **(C)** XRD spectrum; **(D)** Zeta potential (n = 3); **(E)** Adsorption-desorption isotherm and pore diameter distribution measured by BET method; **(F)** XPS spectrum; **(G)** C 1s XPS spectrum; **(H)** O 1s XPS spectrum; **(I)** Si 2p XPS spectrum. The values are expressed as the mean ± standard deviation (SD), for inter-group comparison, ***P < 0.001.

A scanning electron microscope (SEM) was used to observe the morphology of the AC-1@MXene heterojunction. As shown in **Figure 2A** and **Figure S1A-B**, the AC-1 nanoparticles exhibited an irregular morphology with an average diameter of 320 ± 76 nm. Monolayer MXene nanosheets showed a well-defined lamellar structure with an average diameter of 1800 ± 802 nm. The hierarchical lamellar architecture of MXene serves as an ideal platform for the homogeneous adhesion of AC-1 particles. EDS was further performed to verify the element composition, revealing the simultaneous presence of Si, Ti, B, C, N, and O elements in the AC-1@MXene heterojunction.

The chemical structure of the AC-1@MXene heterojunction formed by APTES-COF-1 (AC-1) and MXene was analyzed using FT-IR spectroscopy (**Figure 2B**). By comparing the infrared spectra of MXene and AC-1, several distinct features were identified in the FT-IR curve of AC-1@MXene: The peak at 600 cm^-1^ corresponds to the stretching vibration of the C-Ti bond in MXene, matching with known positions in MXene structures. The pronounced peak at 1350 cm^-1^ is assigned to the C-N bond vibration in the AC-1 framework. Additionally, consecutive absorption peaks in the range of 1000-1100 cm^-1^ indicate the presence of C-O and C-N bonds. All of these peaks indicate characteristics of the AC-1 structure, consistent with the typical chemical composition of AC-1 according to the literature 43,44. XRD analysis (**Figure 2C**) revealed four distinct diffraction peaks at 2θ values of 7.8°, 11.4°, 18.7°, and 27.9° in AC-1@MXene, corresponding to the (100), (110), (201), and (002) crystal planes of the AC-1 component, respectively. These diffraction patterns are consistent with a hexagonal unit cell structure, characteristic of well-ordered AC-1 materials. Notably, the XRD pattern of AC-1@MXene also retained the characteristic (002) diffraction peak of MXene at approximately 8.5° 45-47, indicating that the in-situ growth process did not compromise the crystalline structure of the MXene substrate. These results collectively confirm the successful fabrication of a heterostructured AC-1@MXene composite with preserved structural integrity.

Zeta potential measurements (**Figure 2D**) were performed. The AC-1 component exhibited a zeta potential of -25 ± 0.5 mV, the negative zeta potential of AC-1 under neutral conditions can be attributed to the dominant contribution of inherent negative charges from the COF-1 framework, which contain deprotonated oxygen-containing functional groups (such as hydroxyl and boronic acid groups), that significantly outweigh the limited positive charges introduced by partial APTES functionalization. Since APTES modification is typically incomplete with less than 100% surface coverage, the underlying COF-1 surface properties remain predominant, resulting in a net negative surface charge even in the presence of amino groups. Pristine MXene displayed a negative zeta potential of -34 ± 0.2 mV, arising from its surface termination groups (e.g., -OH, -O^-^). Notably, the AC-1@MXene composite exhibited a zeta potential of -38 ± 0.4 mV, which is more negative than either individual component. This enhancement is attributed to the formation of a heterostructure interface 48, where charge redistribution occurs due to the alignment of Fermi levels between AC-1 and MXene, resulting in increased surface charge density. This high-density negative charge structure has the potential to regulate the polarization of macrophages, which may be conducive to wound healing 49.

For photocatalytic nanozyme materials applied in wound healing, specific surface area and pore structure are critical parameters that determine both catalytic efficiency and enzymatic activity. Analysis of the N₂ adsorption-desorption isotherms (**Figure 2E**, **S2**) via BET measurements revealed a type IV-H3 isotherm with a distinct hysteresis loop, characteristic of a mesoporous adsorption structure. BET analysis further demonstrated that the AC-1@MXene composite exhibits a specific surface area of 36.27 ± 0.05 m^2^/g and an average pore diameter of 2.89 ± 0.01 nm. Compared to pristine AC-1 (40.47 ± 0.06 m²/g, 2.04 ± 0.01 nm), the composite retains substantial porosity with only a 10.4% reduction in surface area, indicating that the in-situ growth process effectively preserves the catalytic framework essential for both photocatalytic and nanozyme functions. This moderate decrease results from the strategic integration of MXene nanosheets, which provide additional catalytic sites and enhanced electron transfer pathways, while the slight structural densification optimizes the synergistic interaction between components. The increase in average pore diameter from 2.04 to 2.89 nm facilitates improved substrate accessibility and product diffusion, crucial for efficient enzymatic turnover. The expanded mesoporous channels create an optimal microenvironment that mimics natural enzyme active sites while providing enhanced mass transfer capabilities.

XPS analysis (**Figure 2F**) revealed characteristic elemental peaks of the composite. Notably, the intensities of F 1s and Ti 2p signals were significantly attenuated in AC-1@MXene compared to pristine MXene, indicating surface coverage by COF particles. High-resolution XPS spectra of C 1s and O 1s (**Figure 2G-H**) showed shifts in C-Ti bonding peaks and the emergence of a prominent Si-O peak (103.5 eV), confirming the formation of interfacial interactions between COF and MXene. The observed shifts in C 1s and O 1s binding energies further indicated altered chemical environments due to hybridization between the two components. Deconvolution of the Si 2p spectrum (**Figure 2I**) revealed a prominent Si 2p_3/2_ peak at 103.5 eV, attributed to Si-O bonds in the APTES framework of the COF structure. Furthermore, X-ray photoelectron spectroscopy (XPS) analysis of the Ti 2p region (**Figure S3**) reveals distinct spin-orbit splitting into Ti 2p_1/2_ and Ti 2p_3/2_ doublets, confirming the coexistence of multiple titanium oxidation states (Ti^4+^/Ti^3+^) in both pristine MXene and AC-1@MXene composite. The relative contents of Ti^3+^ and Ti^4+^ were quantitatively determined through deconvolution and integration of the Ti 2p_3/2_ component peak areas. The results demonstrate that pristine MXene contains 12.83% Ti^3+^ and 87.17% Ti^4+^, while the AC-1@MXene composite exhibits 27.64% Ti^3+^ and 72.36% Ti^4+^. The significant increase in Ti^3+^ content to approximately 28% in the AC-1@MXene composite establishes enhanced electron transfer pathways through Ti^3+^ defect states and associated oxygen vacancy defects, which collectively promote charge separation and surface redox activity, consistent with previous literature reports 50-52. The appearance of new interfacial bonding features and systematic peak shifts collectively confirms the successful fabrication of AC-1@MXene with strong interfacial interactions between AC-1 and MXene.

### Evaluation of photoelectric conversion and nano-enzyme activity

In this study, to enhance the visible light-activated antioxidant nanozyme activity of AC-1, we incorporated two-dimensional MXene nanosheets with excellent electrical conductivity and visible light photocatalytic properties. Owing to the heterojunction structure formed between MXene and AC-1, the composite demonstrated exceptional generation of photogenerated electron-hole pairs and efficient electron transfer, thereby significantly boosting its antioxidant nanozyme activity. To further characterize the photocatalytic performance of AC-1@MXene, we conducted electrochemical measurements.

**Figure 3:**
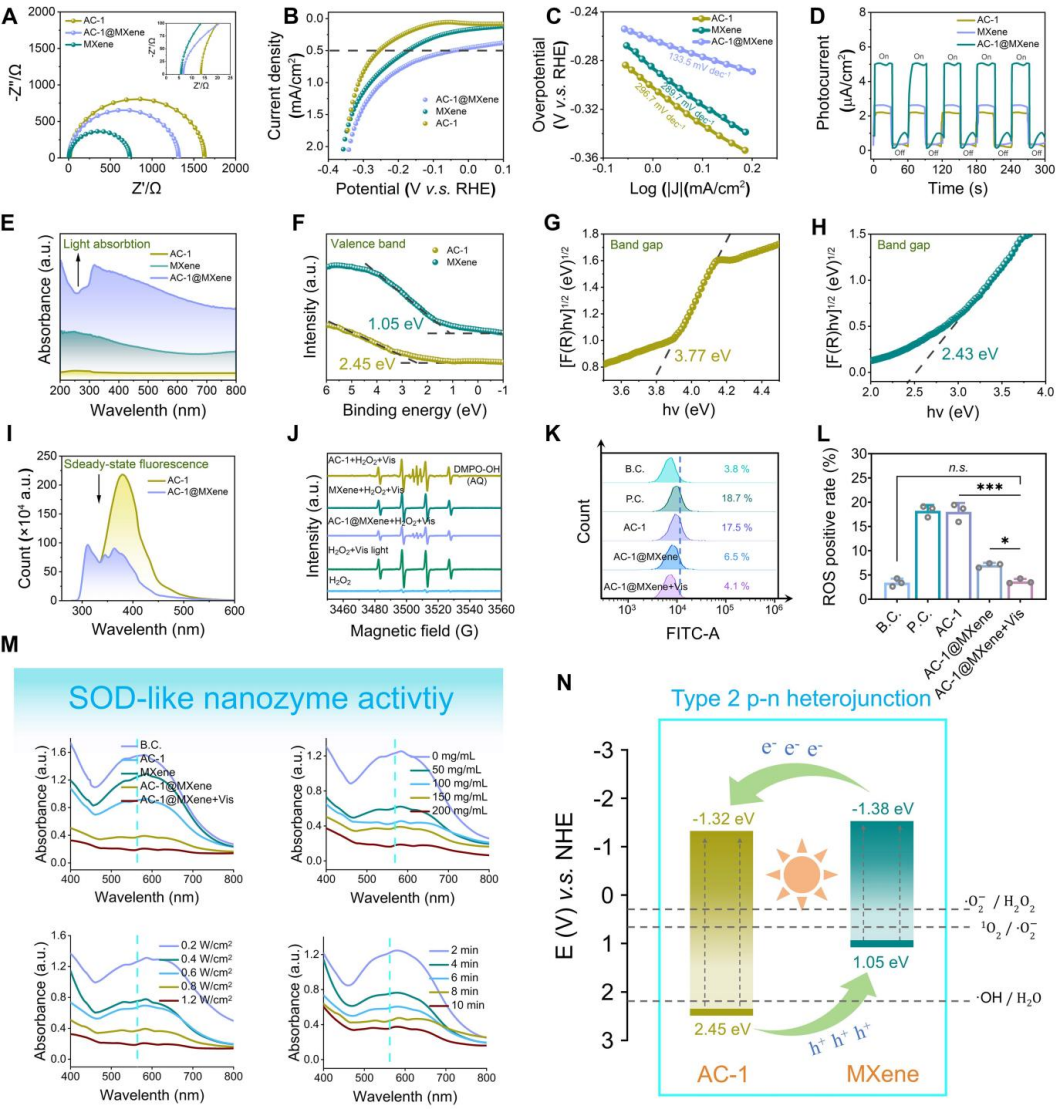
** The anti-oxidative activity and mechanism of AC-1@MXene nanozyme. (A)** AC impedance spectrum; **(B)** Linear sweep voltammetry (LSV) curves; **(C)** Tafel slope; **(D)** Transient photocurrent i-t curves; **(E)** UV-Vis diffuse reflectance spectra; **(F)** Valence band XPS spectrum; **(G)** Bandgap of AC-1 nanoparticles; **(H)** Bandgap of monolayer MXene nanosheets; **(I)** Photoluminescence spectrum; **(J)** EPR spectrum for detecting the ·OH; **(K)** Cell-based anti-oxidative test by flow cytometry; **(L)** Quantitative results of flow cytometry (n = 3); **(M)** *In vitro* anti-oxidative test by NBT assay; **(N)** Schematic illustration of the heterojunction structure and mechanism. The values are expressed as the mean ± standard deviation (SD), for inter-group comparison, *n.s.* indicates no significance, *P < 0.05 and ***P < 0.001.

EIS (**Figure 3A**) revealed that the charge transfer resistance of AC-1@MXene decreased from 1639 Ω (AC-1) to 1270 Ω, reduced by approximately 22.5% that indicating improved interfacial charge transfer efficiency, this will be beneficial to the migration of photogenerated carriers. LSV **(Figure 3B)** showed that under visible light irradiation and an applied reverse bias, AC-1@MXene exhibited a transient photocurrent density exceeding 5.13 ± 0.8 mA/cm^2^, more than twice that of pristine AC-1 (< 2.50 mA/cm^2^). Tafel analysis **(Figure 3C)** yielded a slope of 133.5 ± 0.7 mV dec^-1^ for AC-1@MXene, significantly lower than that of AC-1 (215.6 ± 0.8 mV dec^-1^), indicating superior reaction kinetics and electrocatalytic activity. Cyclic photocurrent measurements (**Figure 3D**) demonstrated excellent photostability of AC-1@MXene, with consistent on/off photocurrent responses over five consecutive cycles. The enhanced photocurrent density of AC-1@MXene can be attributed to the formation of type-II heterojunctions, which facilitate efficient charge separation and suppress electron-hole recombination. These results highlight the synergistic effect of AC-1 and MXene in promoting photoelectrochemical performance, making AC-1@MXene a promising candidate for antioxidant applications 40,47. The superior photoelectric response of AC-1@MXene can be attributed to the synergistic effect of MXene incorporation, and the heterojunction interface formed by them has significantly improved charge separation efficiency. UV-DRS (**Figure 3E**) revealed that AC-1@MXene exhibits the highest absorbance across the visible spectrum, indicating a significantly enhanced visible-light response capability. Valence band of XPS analysis and bandgap analysis (**Figure 3F-H**) displayed the bandgap energies of AC-1 and MXene to be 3.77 eV and 2.43 eV, respectively. Both AC-1 and MXene were identified as direct bandgap semiconductors with valence band maxima (VBM) of 2.45 eV and 1.05 eV, respectively 43. These data support the formation of a type-II heterojunction (**Figure 3N**), wherein photogenerated electrons transfer from the higher conduction band (CB) of MXene (-1.38 eV) to that of AC-1 (-1.32 eV) under visible light irradiation, while holes migrate in the opposite direction. Comparison with standard reduction potentials 53 confirms that the heterojunction is sufficiently negative to reduce reactive oxygen species (ROS) such as •OH (2.38 eV vs. NHE) and •O_2_^-^ (0.36 eV vs. NHE) providing a theoretical basis for its antioxidant activity 54,55. The reduced bandgap of AC-1@MXene (3.18 eV vs. 3.77 eV for AC-1) enables absorption of lower-energy photons, extending its photo-response into the visible region (**Figure S4**).

PL spectroscopy (**Figure 3I**) further corroborated efficient charge separation in AC-1@MXene, as evidenced by a 62 ± 5% quenching of PL intensity relative to pristine AC-1. The observed redshift in emission peak position indicates the formation of interfacial charge transfer states between AC-1 and MXene, which facilitate charge separation and modify the electronic structure of the heterojunction. Collectively, these results underscore the pivotal role of heterojunction engineering in enhancing the photoelectrochemical performance of AC-1@MXene for ROS scavenging applications.

The antioxidant activity of the nanocomposites was evaluated using ultraviolet spectrophotometry to measure superoxide dismutase (SOD)-like activity (**Figure 3M**). AC-1@MXene exhibited significantly higher antioxidant activity compared to AC-1and pristine MXene, achieving optimal performance at a concentration of 100 mg/mL in aqueous solution under visible light irradiation (0.8 W/cm^2^) for 8 min. AC-1@MXene effectively converted superoxide anions (·O_2_^-^) to hydrogen peroxide (H_2_O_2_), as confirmed by the increase in absorbance at 560 nm. Combined with peroxidase CAT-like activity assays (**Figure S5**), AC-1@MXene was shown to simultaneously decompose H_2_O_2_ into water and oxygen, thereby preventing the generation of hydroxyl radicals (·OH).

To evaluate the enzymatic catalytic performance of AC-1@MXene, substrate concentrations were measured using a spectrophotometer and nanozyme activity curves were plotted based on the Michaelis-Menten equation. Through double reciprocal calculations and enzyme kinetics assays (**Figure S6**), the maximum reaction rates (V_max_) of AC-1@MXene for superoxide dismutase (SOD)-like and catalase (CAT)-like enzymatic activities were determined to be 0.07388 μM/min/mg and 2.565 μM/min/mg, respectively. The corresponding Michaelis constants (K_m_) were 0.03422 mM and 67.8 mM for SOD-like and CAT-like activities, respectively. Analysis of these kinetic parameters revealed that the SOD-like nanozyme exhibits significantly higher substrate affinity (·O_2_^-^) as indicated by the smaller Km value, while the CAT-like activity shows relatively weaker substrate affinity for H_2_O_2_.

The specificity constants (K_cat_/K_m_), which quantify catalytic efficiency, were calculated to be 9822.8 and 6.8161 for the SOD-like and CAT-like activities, respectively, demonstrating that the nanozyme possesses substantially stronger catalytic efficiency in SOD-like reactions. This kinetic profile enables the SOD-like component to preferentially initiate reactions during ROS scavenging, rapidly dismutating ·O_2_^-^ into H_2_O_2_, which is subsequently decomposed efficiently by the CAT-like component. Through this temporal sequential synergy, the dual enzymatic activities achieve stepwise ROS scavenging, thereby maintaining cellular redox homeostasis. A comprehensive comparison of enzymatic kinetic parameters between AC-1 and AC-1@MXene (**Table S1**) further demonstrates that the composite nanozyme exhibits superior enzymatic catalytic efficiency compared to AC-1 alone, highlighting the beneficial effect of MXene integration on overall catalytic performance.

EPR spectroscopy (**Figure 3J**) provided direct evidence for the scavenging of •OH radicals by AC-1@MXene, further corroborating its antioxidant efficacy. This synergistic enzyme-like activity of AC-1@MXene enhances the antioxidant defense system *in vivo* and effectively mitigates oxidative stress in chronic wound microenvironments, highlighting its potential for treating chronic skin wounds 56,57.

To validate the *in vitro* antioxidant performance of AC-1@MXene, flow cytometry assays were conducted using L929 cells (**Figure 3K-L**). Under both blank and positive control conditions, AC-1@MXene exhibited excellent antioxidant activity upon visible light irradiation, with results comparable to those of the blank control. This indicates that the synthesis of AC-1@MXene holds significant biological significance in regulating the cellular microenvironment under oxidative stress. Furthermore, the migration viability of cells was examined under oxidative stress models (**Figure S7-8**). The results also demonstrated that AC-1@MXene under antioxidant stress effectively inhibited strong oxidation and maintained good cell viability under light 55.

As a photocatalytic nanozyme, AC-1@MXene provides abundant reactive sites for light-enhanced catalytic decomposition of harmful ROS species, exhibiting intrinsic enzyme-like activity (such as catalase and superoxide dismutase mimicking) that is significantly amplified under light irradiation. This photo-enhanced nanozyme effect enables accelerated conversion of cytotoxic H_2_O_2_ and ·O_2_^-^ into harmless H_2_O and O_2_, providing superior antioxidant protection compared to conventional antioxidants. The well-maintained high surface area ensures maximum exposure of both photocatalytic and enzymatic active sites, while the optimized mesoporous architecture facilitates rapid substrate recognition, binding, and product release - mimicking the efficiency of natural enzymatic processes while offering the controllability of photocatalytic systems 46,58.

### Preparation and characterization of ACMGM hydrogel microneedles

**Figure 4:**
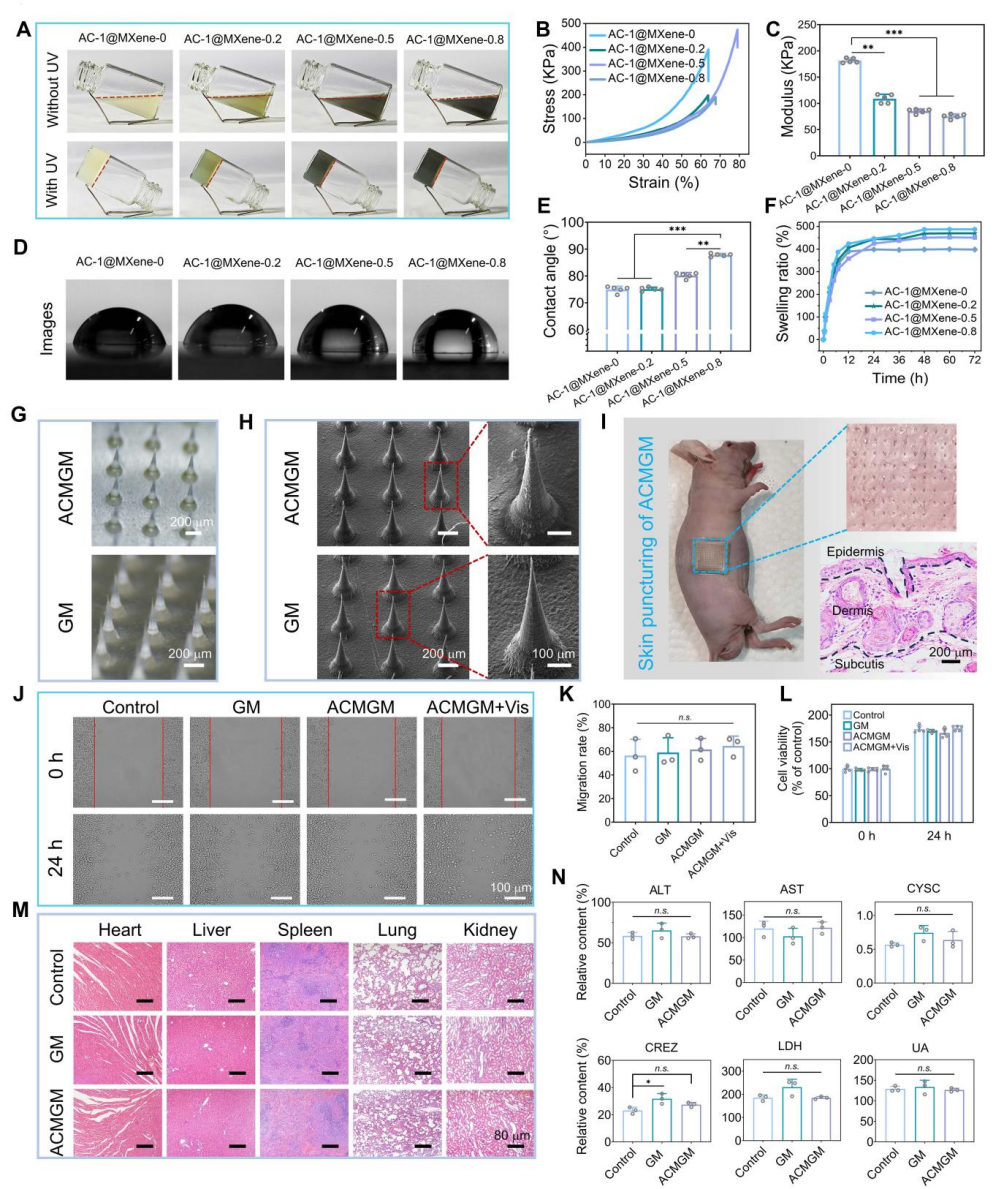
** The fabrication of AC-1@MXene-laden microneedles with double-layer structure and desirable biocompatibility. (A)** Four kinds of hydrogels with varied proportion of GelMA and AC-1@MXene were prepared by UV crosslinking; **(B)** Stress-strain curves; **(C)** Quantitative results of Young's modulus (n = 5); **(D)** Images of water contact angle (WCA); **(E)** Quantitative results of WCA (n = 5); **(F)** Swelling dynamics; **(G)** Optical images of ACMGM and GM microneedles, scale bar: 200 μm; **(H)** SEM images, scale bar: 100 and 200 μm; **(I)** Skin puncture test and H&E staining images of the skin puncture; **(J)** A scratching test was performed to evaluate cell migration ability, scale bar: 100 μm; **(K)** Quantitative results of cell migration rate (n = 3); **(L)** Cell viability (n = 3); **(M)** H&E staining images of the organs, scale bar: 80 µm; **(N)** Quantitative results of blood biochemical tests (n = 3). The values are expressed as the mean ± standard deviation (SD), for inter-group comparison, *n.s.* indicates no significance, *P < 0.05, **P < 0.01 and ***P < 0.001.

Furthermore, AC-1@MXene was incorporated into biocompatible GelMA to fabricate AC-1@MXene-GelMA (ACMGM) hydrogel microneedles. To investigate the influence of micro-nano particle incorporation on hydrogel performance, AC-1@MXene was added at mass percentages of 0%, 0.2%, 0.5%, and 0.8% to 15% GelMA solutions. Gelation was initiated through ultraviolet photopolymerization. As shown in **Figure 4A**, successful gelation was achieved for all hydrogel formulations across the tested AC-1@MXene concentration range.

Since human skin undergoes stretching and contraction during movement, wound dressings must possess adequate mechanical properties to accommodate dynamic skin deformation. We evaluated the compressive mechanical properties of the hydrogel materials to verify their mechanical performance. From the stress-strain curves (**Figure 4B-C**), the incorporation of AC-1@MXene altered the mechanical characteristics of the hydrogels. While the elastic modulus gradually decreased with increasing AC-1@MXene content, indicating reduced stiffness, the addition of an optimal amount of AC-1@MXene-0.5 significantly enhanced the hydrogel toughness. This formulation demonstrated greater ultimate fracture strain and improved energy absorption capacity compared to the control group (AC-1@MXene-0), enabling better accommodation of skin deformation during body movement.

To gain deeper insights into the hydrogel properties, we conducted comprehensive physicochemical characterization of the hydrogel system. **Figure 4D-E** shows the surface wettability evaluation of the hydrogels. The results demonstrated that all microneedles maintained hydrophilic characteristics, with the water contact angle gradually increasing as the AC-1@MXene mass ratio increased. This phenomenon can be attributed to the surface composition change where AC-1@MXene nanoparticles, which possess relatively lower hydrophilicity compared to the GelMA matrix, progressively alter the surface properties. The incorporation of nanoparticles may also create micro-nano surface textures and occupy hydrophilic binding sites within the GelMA network, contributing to the observed increase in contact angle while preserving the overall hydrophilic nature.

We further evaluated the swelling behavior of the hydrogels (**Figure 4F**), which reached swelling equilibrium after 48 hours of immersion in water. Compared to AC-1@MXene-0, the AC-1@MXene-incorporated hydrogels exhibited enhanced swelling capacity, which can be attributed to the reduced crosslinking density of the GelMA network caused by physical interference from AC-1@MXene nanoparticles during the photopolymerization process. This correlation was further supported by the mechanical characterization results, where decreased crosslinking density led to increased network porosity and consequently higher water uptake capacity.

To characterize the biodegradability of the materials, a 7-day degradation experiment was conducted using samples of identical size and mass (**Figure S9**). On the 7th day, the degradation rate of AC-1@MXene-0.5 was 17.5 ± 0.8%, while that of AC-1@MXene-0 was approximately 24.1 ± 0.7%. The lower degradation rate observed for AC-1@MXene-incorporated hydrogels may be attributed to the relatively stable inorganic MXene component within the composite matrix, which degrades more slowly than the organic GelMA network, thereby reducing the overall mass loss rate of the composite system.

In summary, our results demonstrate a significant dose-dependent effect of AC-1@MXene on the overall performance of ACMGM hydrogels. These physicochemical properties play crucial roles in determining the biomedical applicability of the composite system. Therefore, it is essential to select the optimally balanced formulation for subsequent studies to enhance research efficiency. After comprehensive evaluation of mechanical properties, swelling behavior, and degradation characteristics, we selected the AC-1@MXene-0.5 hydrogel as the optimal substrate for follow-up experiments 59.

Double-layer microneedles were fabricated using a custom-designed mold. Each microneedle (MN) featured a conical geometry with a height of 800 μm, a base diameter of 250 μm, and an inter-MN spacing of 400 μm, as illustrated in **Figure 4G**. The control hydrogel without AC-1@MXene was designated as GelMA microneedle (GM), whereas the hydrogel containing 0.5% AC-1@MXene was labeled as ACMGM microneedle. The tip layer of the bilayer microneedles exhibited a characteristic grayish-black coloration due to AC-1@MXene incorporation. SEM images (**Figure 4H**) revealed that the irregular protrusions on the tip surface corresponded to the distribution sites of AC-1@MXene nanozyme particles. These structural features enable the bilayer microneedles to efficiently deliver the nanozyme system upon penetration into the subcutaneous tissue 60, facilitating targeted antioxidant therapy through the catalytic activity of the AC-1@MXene heterostructure. To evaluate the transdermal efficacy of the microneedles, skin puncture experiments were conducted using BALB/c-nu nude mice (**Figure 4I**), combined with mechanical testing of microneedles (**Figure S10**), under the same condition where the deformation reached 0.5 mm, the force values borne by the two types of microneedles (GM and ACMGM) showed a certain difference, being 37.684 ± 6.156 N and 25.823 ± 4.571 N, and a single microneedle was 0.167 ± 0.05 N and 0.115 ± 0.03 N, respectively. The results demonstrated that the microneedles could readily penetrate the skin barrier, effectively creating microchannels for deep delivery of the nanozyme system and facilitating enhanced wound healing.

### Biocompatibility evaluation

Non-toxicity and excellent biocompatibility are critical evaluation criteria for bioengineered materials. To assess the *in vitro* and *in vivo* biocompatibility of ACMGM microneedles, this study employed mouse fibroblast L929 cells for *in vitro* experiments, including wound-healing scratch assays and CCK-8 cytotoxicity tests.

In the scratch migration assay (**Figures 4J-K**), L929 cells treated with ACMGM extract exhibited proliferation and migration rates comparable to those of the blank control group, with no significant differences observed in the quantitative analysis among all groups, indicating minimal impact on cellular activity. The CCK-8 cytotoxicity test (**Figure 4L**) revealed that both GM and ACMGM microneedles had negligible effects on cell viability over 24 h. Statistical analysis showed no significant differences between the treatment groups and control group at both 0 h and 24 h time points, confirming excellent *in vitro* biocompatibility.

To evaluate the *in vivo* biocompatibility, the microneedle materials were subcutaneously implanted in SD rats for 15 days. Blood samples were collected for biochemical analysis, and histological examinations (H&E staining) were performed on major organs (heart, liver, spleen, lung, kidney). The results are presented in **Figure 4M-N and S11**. Histological observations revealed no significant pathological changes between the experimental and control groups, with no evidence of organ lesions or abnormalities in any treatment group. Biochemical analyses of blood parameters (ALT, AST, CRE, CYS-C, LDH, UA, UREA, Na^+^, K^+^) showed values within normal physiological ranges for all groups, confirming the absence of systemic toxicity 61, These results demonstrate normal organ function with no evidence of hepatotoxicity, cardiotoxicity, nephrotoxicity, or electrolyte imbalance.

Collectively, these comprehensive *in vitro* and *in vivo* studies confirm the excellent biocompatibility of AC-1@MXene-incorporated hydrogel microneedles, supporting their potential for safe biomedical applications.

### Evaluation of wound healing in diabetes

**Figure 5:**
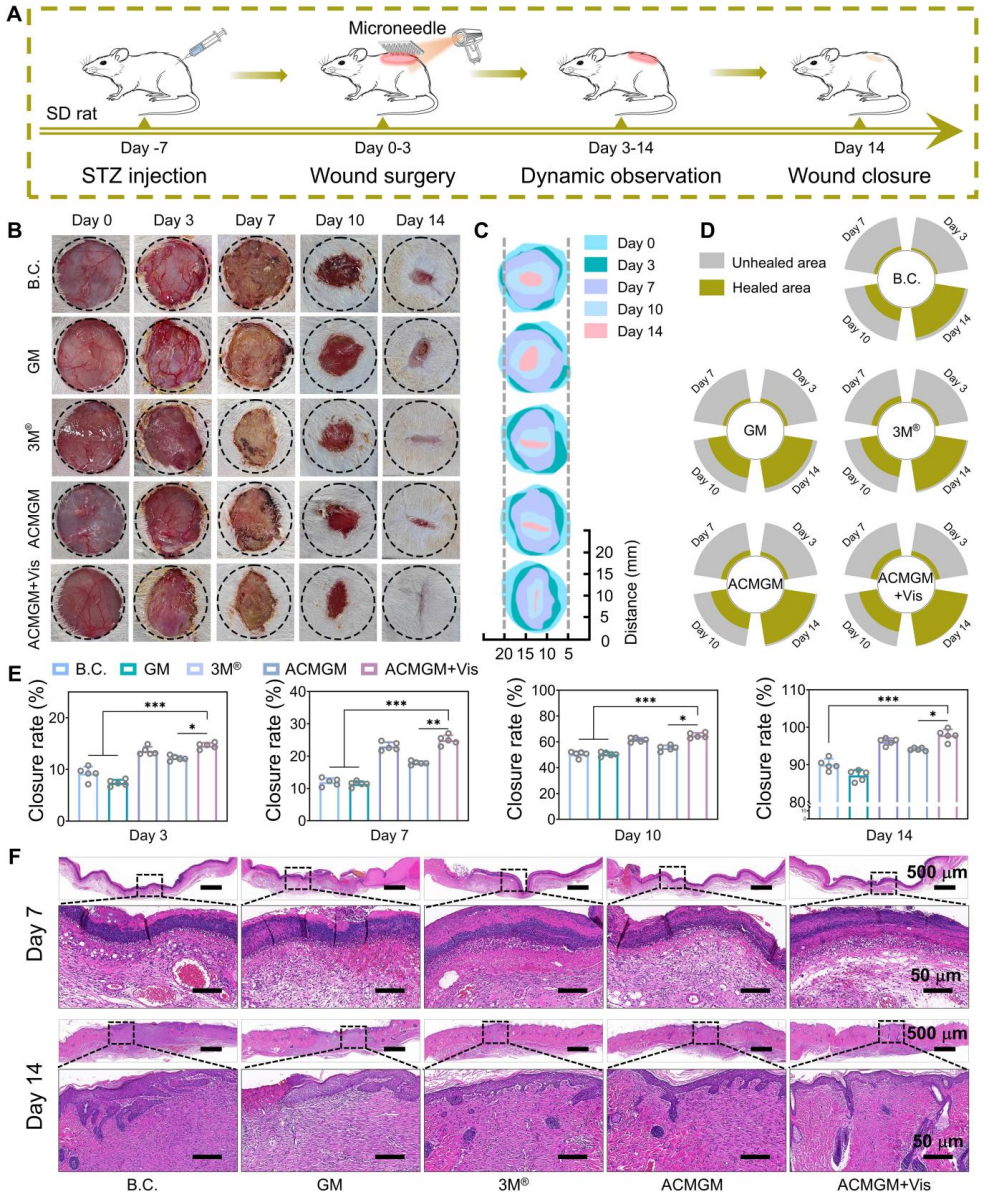
** Diabetic wound healing evaluations *in vivo*. (A)** A diagram showing the animal experiment protocols; **(B-C)** Images of wound sites; **(D)** Wound area of each wound site (n = 1); **(E)** Quantitative result of wound healing rate (n = 5); **(F)** H&E staining images of neo-skin tissue, scale bar: 50 and 500 µm. The values are expressed as the mean ± standard deviation (SD), for inter-group comparison, *P < 0.05, **P < 0.01 and ***P < 0.001.

To comprehensively assess the therapeutic efficacy of ACMGM microneedles in promoting diabetic wound healing, a streptozocin (STZ)-induced diabetic rat model was established by intraperitoneal injection to selectively damage pancreatic β-cells in Sprague-Dawley (SD) rats 62, Seven days post-diabetes induction, the rats were randomly assigned to five experimental groups for treatment: 1) Blank control (B.C.) group with no treatment, 2) GM group treated with pure GelMA microneedles, 3) 3M^®^ commercial dressing group, 4) ACMGM group treated with AC-1@MXene-loaded microneedles, and 5) ACMGM + Vis group treated with AC-1@MXene-loaded microneedles combined with visible light irradiation to enhance nanozyme activity (**Figure 5A**).

Serial wound images were captured on days 0, 3, 7, 10, and 14 post-treatment (**Figure 5B**). Wound healing progress was quantitatively analyzed using image processing software, generating composite visualizations (**Figure 5C-D**) and time-dependent wound closure rate graphs (**Figure 5E**). Statistical analysis revealed that the ACMGM + Vis group exhibited significantly accelerated wound healing compared to all other groups. By day 14, the wound closure rate of the ACMGM + Vis group reached 97.89% ± 0.59%, which was significantly higher than that of the GM group (89.67% ± 0.21%), 3M® group (86.42% ± 1.02%), ACMGM group (95.65% ± 0.48%), and B.C. group (93% ± 0.81%).

These findings demonstrate that ACMGM microneedles effectively promote diabetic wound healing through the catalytic antioxidant activity of the AC-1@MXene nanozyme system. The superior performance of the ACMGM + Vis group suggests that visible light irradiation further enhances the enzymatic activity of the heterostructured nanozymes, leading to improved reactive oxygen species scavenging, reduced oxidative stress, and accelerated tissue regeneration in the challenging diabetic wound microenvironment.

To further validate the wound-healing efficacy and mechanism of ACMGM hydrogel microneedles, we collected newly formed skin tissues at intermediate (day 7) and late (day 14) stages of wound healing for histological analysis. Hematoxylin and eosin (H&E) staining (**Figure 5F**) revealed that, as early as day 7 post-treatment, wounds treated with ACMGM exhibited significantly more abundant and well-organized regenerated epithelial granulation tissue compared to the blank control (B.C.) and other experimental groups. By day 14, while an epithelial layer had formed in all groups, the ACMGM under visible light irradiation (ACMGM + Vis) group demonstrated the most complete wound closure, characterized by extensive neovascularization and the highest density of nascent hair follicles. In contrast, other groups still presented residual unhealed epithelial defects, indicating incomplete tissue repair.

**Figure 6:**
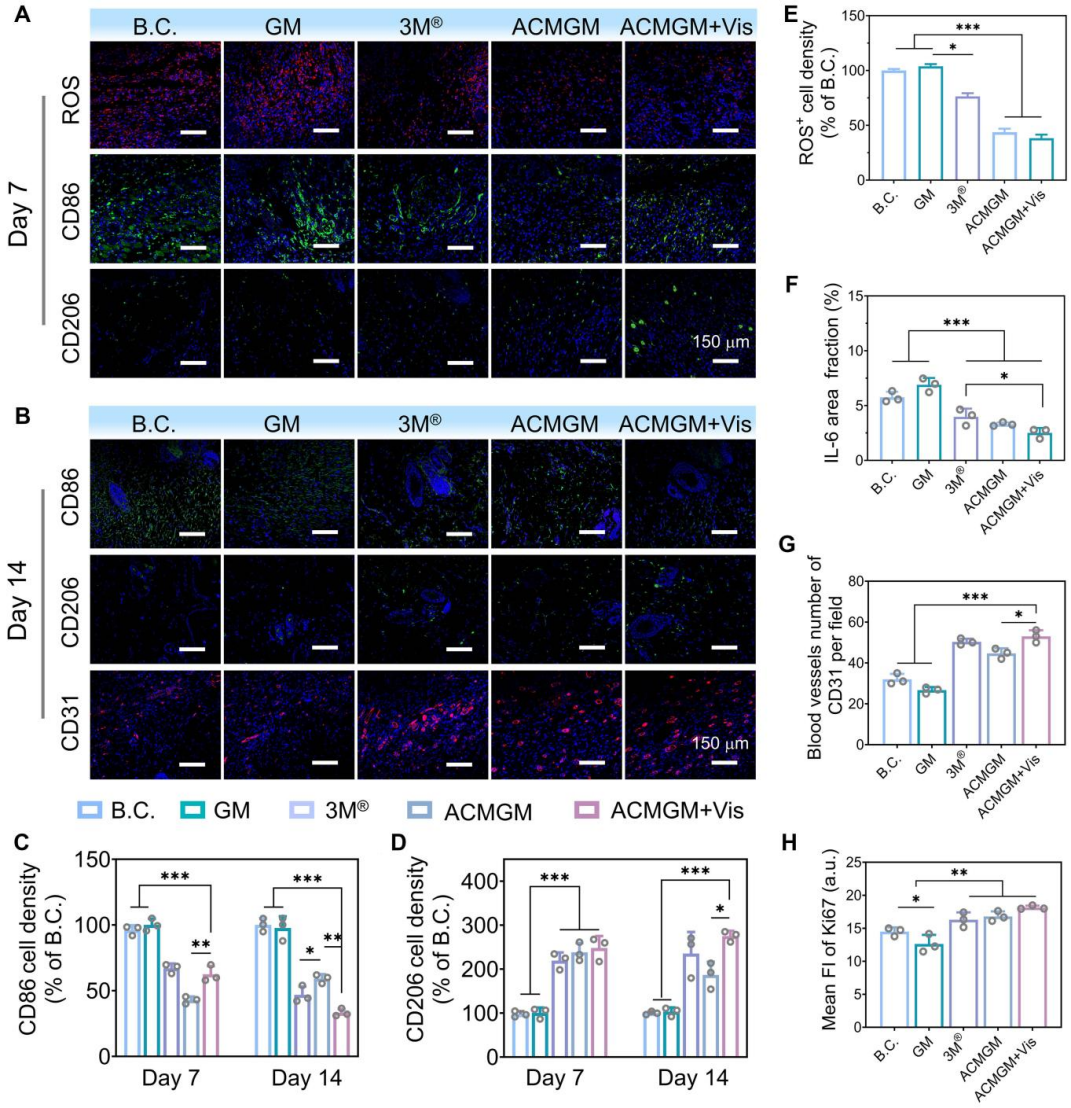
** Mechanism investigation of diabetic wound healing. (A-B)** Immunofluorescence staining of DCFH-DA (ROS probe), CD31, CD86 and CD206 at day7 and day 14, scale bar: 150 µm; **(C-D)** Quantitative results of CD86 and CD206 (n = 3); **(E-F)** Quantitative results of ROS and IL-6 (n = 3); **(G-H)** Quantitative results of CD31 and Ki67 (n = 3). The values are expressed as the mean ± standard deviation (SD), for inter-group comparison, *P < 0.05, **P < 0.01 and ***P < 0.001.

On day 7, the expression levels of ROS and inflammation-related factors (**Figure 6A, C-F, Figure S12, 15**) in newly regenerated tissues were quantitatively assessed. Quantitative analysis revealed that ROS levels in the ACMGM + Vis group were reduced by over 50% compared to the B.C. group, effectively maintaining ROS expression within physiological ranges. These findings highlight the superior performance of the light-enhanced AC-1@MXene nanozyme in alleviating oxidative stress associated with chronic wounds *in vivo*. Histochemical analysis of inflammatory markers revealed that, compared to the B.C. group, the ACMGM + Vis group showed significantly suppressed expression of pro-inflammatory factors (TNF-α, IL-6, and CD86) while exhibiting markedly enhanced expression of the anti-inflammatory factor CD206, indicating that ACMGM, through its light-enhanced antioxidant properties, effectively suppressed inflammation and protected against wound tissue damage and infection.

By day 14, the immunomodulatory efficacy (**Figure 6B-D**) of ACMGM + Vis became more pronounced. CD86 expression in the ACMGM + Vis group was 38.56 ± 0.92% of that in the B.C. group, while CD206 expression reached nearly threefold that of the control. *In vitro* polarization assays with RAW 264.7 macrophages (**Figure S16**) revealed that macrophages in the control group exhibited a small, rounded morphology, whereas those in the LPS + IFN-γ and GM groups displayed an irregular shape with multiple pseudopodia. In contrast, macrophages in the ACMGM group showed a tendency toward an elongated morphology. These *in vitro* findings, combined with the *in vivo* observations in rats demonstrating decreased CD86 fluorescence intensity and increased CD206 fluorescence intensity, indicate that a greater proportion of macrophages in the ACMGM intervention group adopted the M2 phenotype. The reduction in M1-type cells alongside the increase in M2-type cells directly reflects the resolution of inflammation and activation of reparative pathways. In **Figure 6B, G**, the CD31-stained neovascularization images of rat neo-skin show that the number of blood vessels per relative area in the B.C., GM, 3M^®^, ACMGM, and ACMGM + Vis groups is 32 ± 5, 28 ± 2, 47 ± 6, 43 ± 3, and 52 ± 4, respectively. It can also be observed that compared with the control group and the GM group, the neovascular lumens in the other three groups are wider, with diameters reaching 2 - 3 times that of the control group. Furthermore, in comparison with the commercial dressing group (3M^®^ group), the ACMGM group exhibits more uniform and ordered vascular distribution. Collectively, these results demonstrate that nanozymes have significant advantages in promoting angiogenesis. Analysis of Ki67 and collagen staining results (**Figure 6H, Figure S13-15**) revealed that, compared with the blank control (B.C.) group, the ACMGM + Vis group exhibited enhanced cellular proliferative activity (as indicated by an increased Ki67 index) and a significant increase in collagen deposition. These experimental findings collectively confirm that the ACMGM + Vis group demonstrates multi-dimensional superior regulatory efficacy in the process of wound healing.

Collectively, these results systematically validate the therapeutic mechanisms of ACMGM hydrogel microneedles *in vivo*. The light-enhanced AC-1@MXene nanozyme effectively alleviated oxidative stress and inflammatory responses in wound areas while significantly accelerating wound healing through enhanced vascularization, cellular proliferation, and collagen deposition. These findings provide experimental validation for the clinical potential of light-enhanced nanozyme-based wound healing strategies.

## Conclusions

This study successfully developed and characterized a novel double-layer microneedle system incorporating AC-1@MXene heterostructured nanozymes for enhanced diabetic wound healing applications. Through an innovative in-situ growth strategy, AC-1 covalent organic framework nanoparticles were directly synthesized on MXene nanosheets to form hierarchically ordered heterostructures with strong interfacial coupling via hydrogen bonding, N→B coordination interactions, and electrostatic interactions. The resulting AC-1@MXene heterostructure was subsequently immobilized into GelMA microneedle tip arrays through UV photopolymerization, creating the ACMGM microneedle system with tunable physicochemical properties and excellent biocompatibility.

The developed AC-1@MXene heterostructure demonstrated photocatalytic nanozyme activity, exhibiting intrinsic enzyme-like activities that mimic both catalase (CAT) and superoxide dismutase (SOD) with significantly enhanced performance under light irradiation. The heterojunction structure achieved superior photocatalytic quantum efficiency through effective suppression of photogenerated electron-hole pair recombination and accelerated separation of photogenerated carriers. The mesoporous channels within the heterostructure created a biomimetic microenvironment that facilitated efficient substrate diffusion and maximized exposure of catalytically active sites, resulting in enhanced mass transfer efficiency and sustained enzyme-like catalytic cycling. Under visible light irradiation, the system efficiently scavenged harmful reactive oxygen species (ROS) by converting cytotoxic H_2_O_2_ and ·O_2_^-^ into harmless H_2_O and O_2_, maintaining a dynamic ROS balance in chronic wounds while providing superior antioxidant protection compared to traditional antioxidants.

Significant therapeutic advances were achieved through comprehensive *in vivo* evaluation using STZ-induced diabetic SD rats, where ACMGM microneedles demonstrated superior wound closure rates and tissue regeneration quality that nearly surpassed commercial 3M^®^ wound dressings. The treatment mechanism was elucidated through multiple pathways: enhanced antioxidant capacity via light-activated nanozyme catalysis, anti-inflammatory effects through ROS reduction, promotion of M2 macrophage polarization toward tissue repair phenotype, and stimulation of collagen deposition as well as revascularization for improved tissue remodeling. The system maintained stable light-activated nanozyme activity in the wound microenvironment, precisely regulating local biochemical processes and accelerating wound healing through cascade enzymatic reactions.

These findings establish the translational potential of AC-1@MXene-based microneedle systems, highlighting their utility not only in diabetic wound care but also in managing other oxidative stress-related wound healing challenges, thereby opening new avenues for advanced nanozyme-mediated therapeutic interventions.

## Supplementary Material

Supplementary methods and figures.

## References

[1] Armstrong DG, Tan TW, Boulton AJM, Bus SA (2023). Diabetic Foot Ulcers: A Review. JAMA.

[2] Mo J, Huang Y, Wang Q, Zhong H, Zhai Z, Nong Y (2023). Autologous wound margin point columnar full-thickness skin grafting combined with negative pressure wound therapy improves wound healing in refractory diabetic foot ulcers. Int Wound J.

[3] Li M, Jafari H, Okoro OV, Nie L, Shavandi A (2025). Nanozymes as catalysts for accelerated healing of diabetic wounds. Cell Biomater.

[4] Uberoi A, McCready-Vangi A, Grice EA (2024). The wound microbiota: microbial mechanisms of impaired wound healing and infection. Nat Rev Microbiol.

[5] Qi X, Cai E, Xiang Y, Zhang C, Ge X, Wang J (2023). An Immunomodulatory Hydrogel by Hyperthermia-Assisted Self-Cascade Glucose Depletion and ROS Scavenging for Diabetic Foot Ulcer Wound Therapeutics. Adv Mater.

[6] Wang J, Wu Z, Ma X, Huang Z, Dong H, Zhang J (2025). NIR-II emissive biohybrid nanovesicles as mild-temperature photothermal antibiofilm agents against acute bacterial skin and skin-structure infections. Interdiscip Med.

[7] Guo Y, Ding S, Shang C, Zhang C, Li M, Zhang Q (2024). Multifunctional PtCuTe Nanosheets with Strong ROS Scavenging and ROS-Independent Antibacterial Properties Promote Diabetic Wound Healing. Adv Mater.

[8] Palomeque Chávez JC, McGrath M, Kearney CJ, Browne S, O'Brien FJ (2025). Biomaterial scaffold-based gene delivery for the repair of complex wounds: Challenges, progress, and future perspectives. Cell Biomater.

[9] Li H, Li B, Lv D, Li W, Lu Y, Luo G (2023). Biomaterials releasing drug responsively to promote wound healing via regulation of pathological microenvironment. Adv Drug Del Rev.

[10] Fang T, Ma C, Zhang Z, Sun L, Zheng N (2023). Roxadustat, a HIF-PHD inhibitor with exploitable potential on diabetes-related complications. Front Pharmacol.

[11] Li Y, Wang Y, Ding Y, Fan X, Ye L, Pan Q (2024). A Double Network Composite Hydrogel with Self-Regulating Cu(2+)/Luteolin Release and Mechanical Modulation for Enhanced Wound Healing. ACS Nano.

[12] Qi XL, Ge XX, Chen XJ, Cai ERY, Xiang YJ, Xu HB (2024). An Immunoregulation Hydrogel with Controlled Hyperthermia-Augmented Oxygenation and ROS Scavenging for Treating Diabetic Foot Ulcers. Adv Funct Mater.

[13] Zhou Z, Ma T, Zhang H, Chheda S, Li H, Wang K (2024). Carbon dioxide capture from open air using covalent organic frameworks. Nature.

[14] Cote AP, Benin AI, Ockwig NW, O'Keeffe M, Matzger AJ, Yaghi OM (2005). Porous, crystalline, covalent organic frameworks. Science.

[15] Zhu Y, Chen L, Pan J, Jiang S, Wang J, Zhang G (2025). Recent advances in COF-derived carbon materials: Synthesis, properties, and applications. Prog Mater Sci.

[16] Li T, Pan Y, Shao B, Zhang X, Wu T, He Q (2023). Covalent-Organic Framework (COF)-Core-Shell Composites: Classification, Synthesis, Properties, and Applications. Adv Funct Mater.

[17] Jin N, Wu J, Ye S, Xue J, Meng T, Hu L (2024). Injectable Dynamic ROS-Responsive COF-Modified Microalgae Gels for In Vivo bFGF Delivery to Treat Diabetic Wounds. ACS Appl Mater Interfaces.

[18] Yan X, Liu N, Liu W, Zeng J, Liu C, Chen S (2024). Recent advances on COF-based single-atom and dual-atom sites for oxygen catalysis. Chem Commun.

[19] Alsudairy Z, Brown N, Campbell A, Ambus A, Brown B, Smith-Petty K (2023). Covalent organic frameworks in heterogeneous catalysis: recent advances and future perspective. Mater Chem Front.

[20] Wang G-B, Xie K-H, Xu H-P, Wang Y-J, Zhao F, Geng Y (2022). Covalent organic frameworks and their composites as multifunctional photocatalysts for efficient visible-light induced organic transformations. Coord Chem Rev.

[21] Zhang M, Lu M, Lang ZL, Liu J, Liu M, Chang JN (2020). Semiconductor/Covalent-Organic-Framework Z-Scheme Heterojunctions for Artificial Photosynthesis. Angew Chem Int Ed Engl.

[22] Geng C, He S, Yu S, Johnson HM, Shi H, Chen Y (2024). Achieving Clearance of Drug-Resistant Bacterial Infection and Rapid Cutaneous Wound Regeneration Using an ROS-Balancing-Engineered Heterojunction. Adv Mater.

[23] Wu X, Tan L, Chen G, Kang J, Wang G (2024). g-C3N4-based S-scheme heterojunction photocatalysts. Sci China Mater.

[24] Wang Q, Zhou H, Qian J, Xue B, Du H, Hao D (2024). Ti3C2-assisted construction of Z-scheme MIL-88A(Fe)/Ti3C2/RF heterojunction: Multifunctional photocatalysis-in-situ-self-Fenton catalyst. J Mater Sci Technol.

[25] Lee D-E, Danish M, Jo W-K (2024). A review of the interfacial chemistry of Ti3C2 MXene-coordinated nanocomposites for photocatalytic green H2 evolution. Coord Chem Rev.

[26] Li B, Yang W, Shu R, Yang H, Yang F, Dai W (2024). Antibacterial and Angiogenic (2A) Bio-Heterojunctions Facilitate Infectious Ischemic Wound Regeneration via an Endogenous-Exogenous Bistimulatory Strategy. Adv Mater.

[27] You W, Cai Z, Xiao F, Zhao J, Wang G, Wang W (2025). Biomolecular Microneedle Initiates Fe3O4/MXene Heterojunction-Mediated Nanozyme-Like Reactions and Bacterial Ferroptosis to Repair Diabetic Wounds. Adv Sci.

[28] Zheng H, Wang SQ, Cheng F, He XW, Liu ZX, Wang WY (2021). Bioactive anti-inflammatory, antibacterial, conductive multifunctional scaffold based on MXene@CeO2 nanocomposites for infection-impaired skin multimodal therapy. Chem Eng J.

[29] Wang X, Mu Y, Yang K, Shao K, Cong X, Cao Z (2022). Reversible Regulation of the Reactive Oxygen Species Level Using a Semiconductor Heterojunction. ACS Appl Mater Interfaces.

[30] Liang H, Chen X, Bu Z, Bai Q, Liu J, Tian Q (2024). When nanozymes meet deoxyribonucleic acid: Understanding their interactions and biomedical diagnosis applications. Interdiscip Med.

[31] Ghoreishian SM, Ranjith KS, Park B, Hwang S-K, Hosseini R, Behjatmanesh-Ardakani R (2021). Full-spectrum-responsive Bi2S3@CdS S-scheme heterostructure with intimated ultrathin RGO toward photocatalytic Cr(VI) reduction and H2O2 production: Experimental and DFT studies. Chem Eng J.

[32] Chen Z, Zhang L, Yang Y, Cheng W, Tu L, Wang Z (2025). Photothermal Nanozyme-Encapsulating Microneedles for Synergistic Treatment of Infected Wounds. Adv Funct Mater.

[33] Wang G, Wang W, Chen Z, Hu T, Tu L, Wang X (2024). Photothermal microneedle patch loaded with antimicrobial peptide/MnO2 hybrid nanoparticles for chronic wound healing. Chem Eng J.

[34] Chen Z, Guo Z, Hu T, Huang B, Zheng Q, Du X (2024). Double-layered microneedle patch loaded with bioinspired nano-vaccine for melanoma treatment and wound healing. Int J Biol Macromol.

[35] Zhu Y, Yu X, Liu H, Li J, Gholipourmalekabadi M, Lin K (2024). Strategies of functionalized GelMA-based bioinks for bone regeneration: Recent advances and future perspectives. Bioact Mater.

[36] Duan W, Xu K, Huang S, Gao Y, Guo Y, Shen Q (2024). Nanomaterials-incorporated polymeric microneedles for wound healing applications. Int J Pharm.

[37] Wang Z, Liang X, Wang G, Wang X, Chen Y (2025). Emerging Bioprinting for Wound Healing. Adv Mater.

[38] Mo R, Zhang H, Xu Y, Wu X, Wang S, Dong Z (2023). Transdermal drug delivery via microneedles to mediate wound microenvironment. Adv Drug Deliv Rev.

[39] Zhao ZQ, Liang L, Jing LY, Liu Y, Zhang YH, Shahbazi MA (2023). Microneedles: a novel strategy for wound management. Biomater Sci.

[40] Liu S, Zhang Y, Guo Y, Cheng Z, Yuan M, Xu Z (2025). Step-scheme/Mott-Schottky integrated heteroiunctions in BiFeO(3)/ZnIn(2)S(4)/Ag hollow nanospheres: Facilitating efficient piezo-photocatalytic activation of peroxydisulfate to enhance nizatidine degradation and antibacterial activity. J Colloid Interface Sci.

[41] Zhao Y, Liu Y, Shan J, Xu X, Zhang C, Liu Z (2025). Anti-inflammatory coupled anti-angiogenic airway stent effectively suppresses tracheal in-stents restenosis. J Nanobiotechnol.

[42] Ye P, Yang Y, Liu M, Meng J, Zhao J, Zhao J (2025). Co-Delivery of Morphologically Switchable Au Nanowire and Hemoglobin-Resveratrol Nanoparticles in the Microneedle for Diabetic Wound Healing Therapy. Adv Mater.

[43] Wang RN, Zhang XR, Wang SF, Fu GS, Wang JL (2016). Flatbands in 2D boroxine-linked covalent organic frameworks. Phys Chem Chem Phys.

[44] Naguib M, Kurtoglu M, Presser V, Lu J, Niu J, Heon M (2011). Two-dimensional nanocrystals produced by exfoliation of Ti3 AlC2. Adv Mater.

[45] Yang Y, Liu Q, Zou Y, Tian M, Wang L, Li L (2023). Covalent assembly synthesis of covalent organic framework and MXene based composite for the adsorption of fluoroquinolones. J Environ Chem Eng.

[46] Du Y, Calabro D, Wooler B, Kortunov P, Li Q, Cundy S (2015). One Step Facile Synthesis of Amine-Functionalized COF-1 with Enhanced Hydrostability. Chem Mater.

[47] Zhang Y, Xu X, Liao Q, Wang Q, Han Q, Chen P (2022). New potential of boron-based COFs: the biocompatible COF-1 for reactive oxygen generation and antimicrobial applications. J Mater Chem B.

[48] Sun L, Li M, Gu J, Li Y, Liu J, Li Y (2023). High-zeta-potential accelerates interface charge transfer in lithium anodes via MXene-graphdiyne heterojunction layers. Chem Eng J.

[49] Wang Z, He X, Tang B, Chen X, Dong L, Cheng K (2021). Polarization behavior of bone marrow-derived macrophages on charged P(VDF-TrFE) coatings. Biomater Sci.

[50] Ding L, Wei Y, Wang Y, Chen H, Caro J, Wang H (2017). A Two-Dimensional Lamellar Membrane: MXene Nanosheet Stacks. Angew Chem Int Ed Engl.

[51] Wang H, Qian C, Liu J, Zeng Y, Wang D, Zhou W (2020). Integrating Suitable Linkage of Covalent Organic Frameworks into Covalently Bridged Inorganic/Organic Hybrids toward Efficient Photocatalysis. J Am Chem Soc.

[52] Peng C, Wang H, Yu H, Peng F (2017). (111) TiO2-x/Ti3C2: Synergy of active facets, interfacial charge transfer and Ti3+ doping for enhance photocatalytic activity. Mater Res Bull.

[53] Vatansever F, de Melo WC, Avci P, Vecchio D, Sadasivam M, Gupta A (2013). Antimicrobial strategies centered around reactive oxygen species-bactericidal antibiotics, photodynamic therapy, and beyond. FEMS Microbiol Rev.

[54] Zhang H, Gu H, Huang Y, Wang X, Gao L, Li Q (2024). Rational design of covalent organic frameworks/NaTaO(3) S-scheme heterostructure for enhanced photocatalytic hydrogen evolution. J Colloid Interface Sci.

[55] Ma Y, Lai X, Luo X, Luo Z, Mao L, Zhu H (2024). Multifunctional Silver-Enzyme Nanogels Assembly with Efficient Trienzyme Cascades for Synergistic Diabetic Wound Healing. Adv Funct Mater.

[56] Nosaka Y, Nosaka AY (2017). Generation and Detection of Reactive Oxygen Species in Photocatalysis. Chem Rev.

[57] Li H, Zheng X, Gao Z, Mu T, Liu M, Li J (2025). ROS-Responsive Core-Shell Microneedles Based on Simultaneous Efficient Type I/II Photosensitizers for Photodynamic Against Bacterial Biofilm Infections. Adv Funct Mater.

[58] Zhang G, Li X, Liao Q, Liu Y, Xi K, Huang W (2018). Water-dispersible PEG-curcumin/amine-functionalized covalent organic framework nanocomposites as smart carriers for in vivo drug delivery. Nat Commun.

[59] Zhu M, Zhang H, Zhou Q, Sheng S, Gao Q, Geng Z (2025). Dynamic GelMA/DNA Dual-Network Hydrogels Promote Woven Bone Organoid Formation and Enhance Bone Regeneration. Adv Mater.

[60] Hou Y, Guo X, Ran J, Lu X, Xie C (2025). Conductive polyphenol microneedles coupled with electroacupuncture to accelerate wound healing and alleviate depressive-like behaviors in diabetes. Bioact Mater.

[61] Boehm O, Zur B, Koch A, Tran N, Freyenhagen R, Hartmann M (2007). Clinical chemistry reference database for Wistar rats and C57/BL6 mice. Biol Chem.

[62] Zhou Y, Yang J, Li Y, Shu X, Cai Y, Xu P (2024). Multifunctional nanocomposites mediated novel hydrogel for diabetic wound repair. J Mater Chem B.

