# Supplementary material for: ROS-targeting heterojunction-integrated GelMA microneedles for photo-responsive antioxidative action and accelerated diabetic wound healing
Source: Theranostics. 2025 Sep 12;15(18):9987–10006. doi: 10.7150/thno.120879 (PMC12486989; doi:10.7150/thno.120879)
Supplement: Supplementary file 1 — Supplementary methods and figures. [file thnov15p9987s1.pdf]

Supporting Information

ROS-targeting heterojunction-integrated GelMA microneedles for photo-responsive antioxidative action and accelerated diabetic wound healing

Jinlan Tang[a,b,†], Weijun Liu[c,†], Zhengyao Zhang[d], Ye Yang[a], Wenwen Cheng[a], Xiaoyu Wang[e], Zesheng Chen[b,*], Zijian Wang[b,d,*] and Weikang Hu[a,*]

[a] School of Materials Science and Engineering, Stem Cells and Tissue Engineering Manufacture Center, Hubei University, Wuhan 430062, China

[b] Department of Urology, Cancer Precision Diagnosis and Treatment and Translational Medicine Hubei Engineering Research Center, Zhongnan Hospital of Wuhan University, Wuhan 430071, China

[c] Department of Spine Surgery, Wuhan Fourth Hospital, Wuhan, 430033, China

[d] Department of Biomedical Engineering, Hubei Province Key Laboratory of Allergy and Immune Related Disease, TaiKang Medical School (School of Basic Medical Sciences), Wuhan University, Wuhan 430071, China

[e] Department of Endocrinology, Jiangxi Provincial People's Hospital, The First Affiliated Hospital of Nanchang Medical College, Nanchang 330006, China

† These authors contributed equally to this work.

* Corresponding authors at: huwkang@hubu.edu.cn (W. Hu); zijianwang@whu.edu.cn (Z. Wang); zeshengchen@whu.edu.cn (Z. Chen).

1. Instruments

UV-visible spectrophotometer (UV-3600, Shimadzu, Japan). Fourier Infrared spectrometer (Nicolet iS5, Thermo, USA). X-ray photoelectron spectroscopy (ESCAlab 250xi, Thermo, USA). Field-emission scanning electron microscope (Hitachi Regulus 8100, HITACHI, Japan). X-ray diffractometer (D8 Advance, Bruker, Germany). Electron paramagnetic resonance spectrometer (EMXplus, Bruker, Germany). Particle size and Z potential analyzer (ZS90, Malvern, UK). Specific surface area and pore size analyzer (MIC-2460, Micromeritics, USA). Inverted fluorescence microscope (IX73, HuiNengZhi, China). Water contact angle measuring instrument (SL200KS, Kono, China). Universal material testing machine (HC-CL100N, TianYiShi, China), Xenon lamp source (PLS-SXE300+, BoFeiLai, China). Homogenizer (PLS-LA320A, BoFeiLai, China)

2. Chemical reagents

3-Aminopropyl triethoxysilane (APTES), 1,4-Phenylenediboronic acid (BDBA), 1,3,5-Trimethylbenzene and 1,4-Dioxane were purchased from Aladdin Industrial Corporation (Shanghai, China). MAX (titanium aluminum carbide), hydrofluoric acid, lithium chloride and H_2O_2 solution was obtained from Sinopharm Chemical Reagent Co., Ltd. (Shanghai, China). Gelatin, methacrylic anhydride (MA) and 2-Hydroxy-4-(2-hydroxyethoxy)-2-methylpropiophenone (2959) were obtained from Macklin Chemical Regent Co., Ltd. (Shanghai, China). The microneedle mold was kindly provided by Zhongding Yuxuan New Material Technology Co., Ltd. (Hefei, China). L929 cell was kindly provided by Medical Research Institute of Wuhan University. The reactive oxygen species assay kit was purchased from Beyotime Biotech Co., Ltd. (Shanghai, China). All reagents were used as received.

3. Photoelectrochemical tests

A 5 wt% neutral sodium sulfate (Na_2SO_4) electrolyte was prepared, and carbon papers were put in 3 M nitric acid (HNO_3) and refluxed in an oil bath at 120 °C for a minimum of 4 h to enhance surface hydrophilicity. A homogeneous catalyst ink was prepared by dispersing 5 mg of the nanocomposite material in 1 mL of a 50% (v/v) ethanol-water mixture. Subsequently, 20 μL of a 5 wt% Nafion solution was added as a binder, and the mixture was further sonicated for an additional 30 min to form a stable suspension. Aliquots of 5 μL were carefully pipetted onto the substrate surface, and this process was repeated 10 times. The prepared nanocomposite-modified carbon paper served as the working electrode, a saturated calomel electrode (SCE, +0.241 V vs. SHE) as

the reference electrode, and a platinum wire (99.99%, diameter 1 mm) as the counter electrode. All electrode potentials reported in this study were referenced to the reversible hydrogen electrode (RHE) using the conversion E(RHE) = E(SCE) + 0.241 V + 0.0591 × pH.

1) EIS measurements were performed in the "IMP" mode. The frequency range was set from 100 kHz to 0.1 Hz with an AC amplitude of 10 mV. All measurements were conducted at open-circuit potential (OCP) in the dark to minimize photoinduced effects. The obtained impedance data were analyzed using ZView software, fitting to an appropriate equivalent circuit model.

2) LSV measurements were carried out in the "LSV" mode with a scan rate of 5 mV. All LSV curves were iR-corrected using the solution resistance obtained from EIS measurements. Tafel slopes were derived from the linear region of the LSV curves using the Butler-Volmer equation.

3) Transient photocurrent measurements were performed in the "i-t" mode at a fixed potential of 0.8 V *vs.* RHE. A Xenon lamp equipped with a homogenizer was used as the visible light source. The output current was adjusted to 15 A, and the distance between the light source and the electrode surface was set to 10 cm, resulting in a final light intensity density of approximately 450 W/cm^2 at the electrode surface. The light was cycled on and off every 30 s for a total duration of 300 s to evaluate the stability and reproducibility of the photocurrent response. The resulting photocurrent-time (i-t) curves were analyzed to determine the steady-state photocurrent density and the photo-response efficiency.

4. Hydrogel gelation test

Composite hydrogels with varying crosslinking densities were synthesized by incorporating 15 wt% GelMA-functionalized matrixes with AC-1@MXene nanozyme at mass fractions of 0 wt%, 0.2 wt%, 0.5 wt%, and 0.8 wt%. The formulations were labeled as AC-1@MXene-0, AC-1@MXene-0.2, AC-1@MXene-0.5, and AC-1@MXene-0.8, respectively. To initiate photo-polymerization, 100 μL of photo-initiator (1 wt% in ethanol) was added per gram of the precursor solution. The mixtures were vortexed for 30 s to ensure homogeneous dispersion and then transferred into cylindrical molds (diameter = 10 mm, height = 2 mm). Samples were irradiated under a 365 nm UV lamp for 10 min to induce crosslinking.

5. Water contact angle

For each experimental group, 2 mL of the hydrogel suspension was carefully pipetted and uniformly spread onto glass slides using a glass rod to form a thin film, ensuring the complete absence of air

bubbles. Subsequently, the samples were exposed to a UV lamp for evaporation-induced drying under controlled conditions. To accurately characterize the morphological features of the dried hydrogel films with varying concentrations, the sessile drop method was employed. Specifically, the water contact angle measuring instrument was utilized to capture high-resolution images of the droplet outer profiles immediately after droplet deposition, which were then analyzed for quantitative morphological parameters. This approach enables precise evaluation of the surface topography and wettability characteristics of the hydrogel films.

6. Compression test

The hydrogel precursor mixture was carefully dispensed into a sterile 24-well plate to fabricate hydrogel columnar solids with uniform volumes. For each experimental group, five parallel specimens were prepared to ensure statistical reliability and experimental reproducibility. Prior to the mechanical testing, the diameters of the upper surfaces of the specimens were accurately measured using a digital caliper with an accuracy of ± 0.01 mm. The compression tests were carried out using a universal testing machine. During the tests, the compression fixture was programmed to descend at a constant rate of 1 mm/min to maintain quasi - static loading conditions. Throughout the compression process, the applied force and corresponding displacement were continuously recorded, enabling the generation of stress - strain curves for each specimen. To determine the Young's modulus, the initial linear segment of the stress - strain curve (corresponding to the first 30% strain) was subjected to linear regression analysis. The slope obtained from this linear fitting procedure represented the Young's modulus.

7. Properties of swelling

Four groups of composite hydrogel materials with similar masses were thoroughly dried until a constant mass was achieved to remove all moisture. The initial mass (W_0) of each dried hydrogel sample was accurately measured using an analytical balance (accuracy: ± 0.1 mg). Subsequently, the samples were immersed in excess deionized water at room temperature (25 ± 1 °C) to initiate the swelling process. The mass (W_t) of each hydrogel sample was recorded at specific time intervals: 10 minutes, 1 h, 3 h, 5 h, 7 h, 10 h, 22 h, 24 h, 48 h, and 72 h. The swelling ratio was calculated according to the following formula: Swelling rate = $(W_t - W_0)/W_0 \times 100\%$.

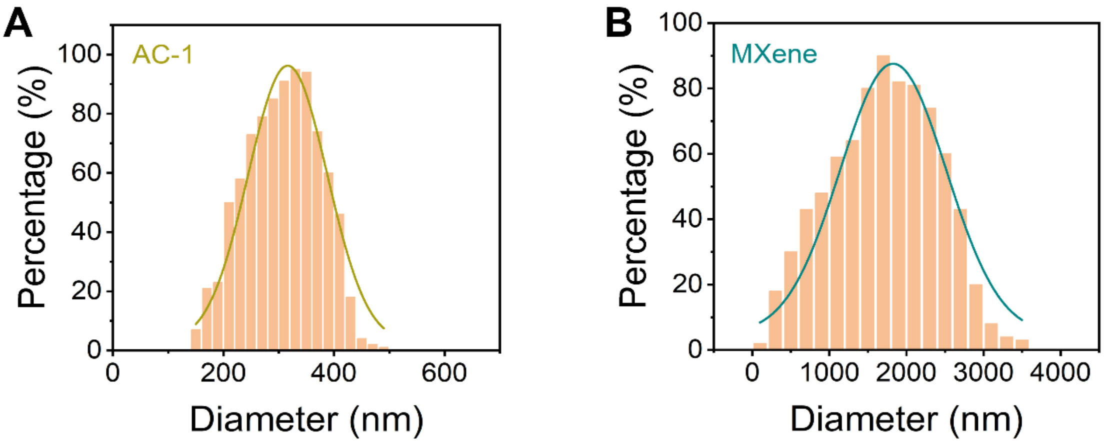

Figure S1. The particle size of the (A) AC-1 and (B) MXene that passed the DLS test, recorded in PBS buffer (pH 7.4, 10 mM).

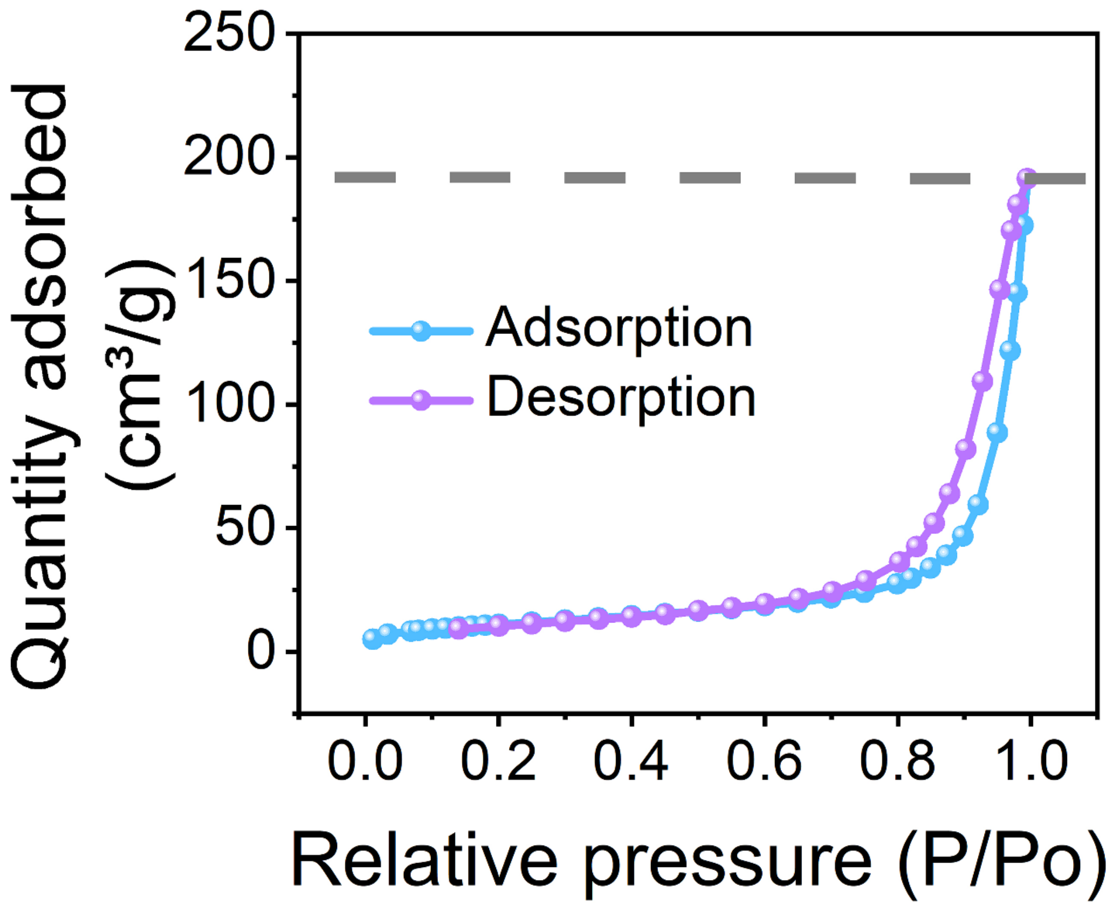

Figure S2. The adsorption/desorption isotherms of AC-1.

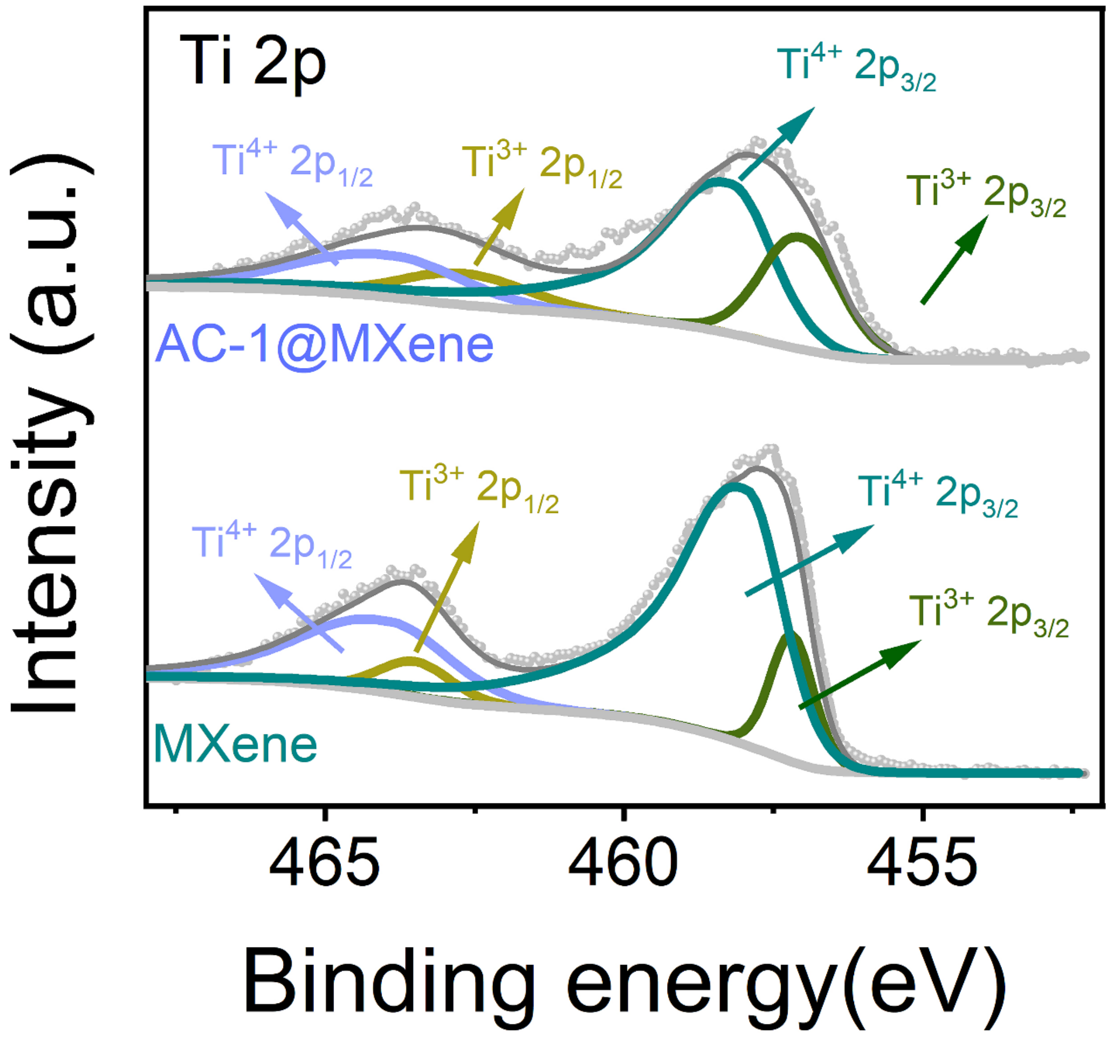

Figure S3. XPS spectrum of Ti 2p signals for AC-1@MXene and MXene.

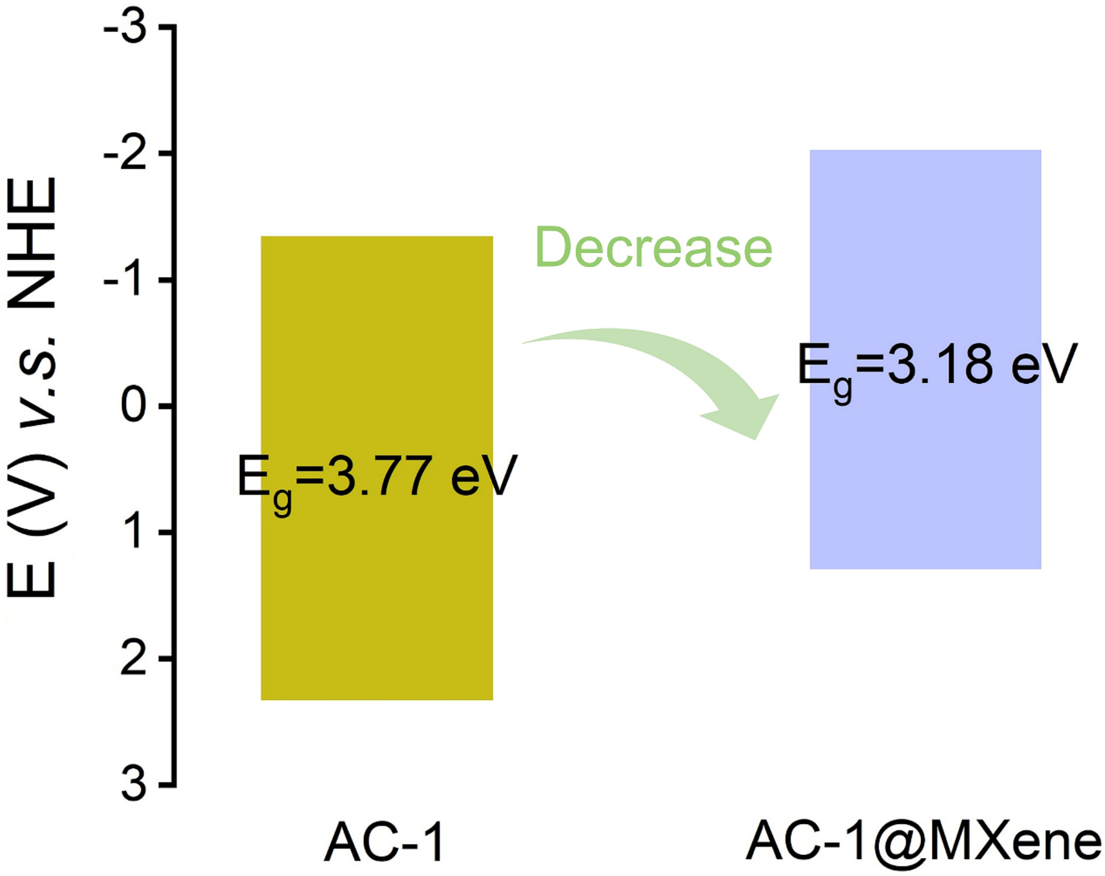

Figure S4. Variation graph of the band gap value of AC-1 and AC-1@MXene.

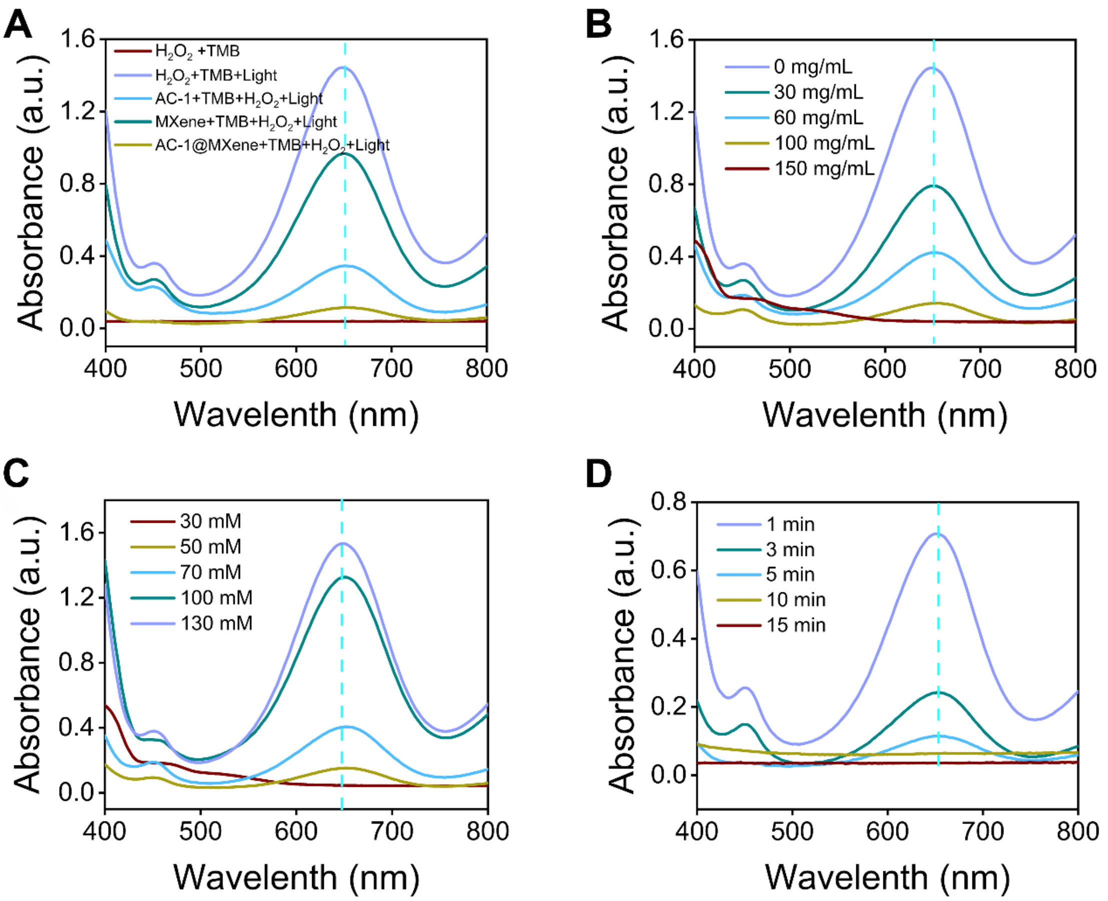

Figure S5. The activity of CAT-like enzymes with TMB as the substrate. (A) Different groups. (B) Different concentrations of AC-1@MXene-0.5. (C) Different concentrations of H_2O_2. (D) Different reaction times.

Table S1 Kinetic parameters of different nanozymes

Nanozyme	Substrates	V_{max} µm/min/mg	K_m mM	K_{cat} min^{-1}	K_{cat}/K_m $mM^{-1} \cdot min^{-1}$
AC-1 @MXene	H_2O_2	2.5654	67.831	462.34	6.8161
	Riboflavin	0.0739	0.0342	335.94	9822.8
AC-1	H_2O_2	1.7831	268.36	464.52	1.7310
	Riboflavin	2.6842	19.347	203.87	10.538

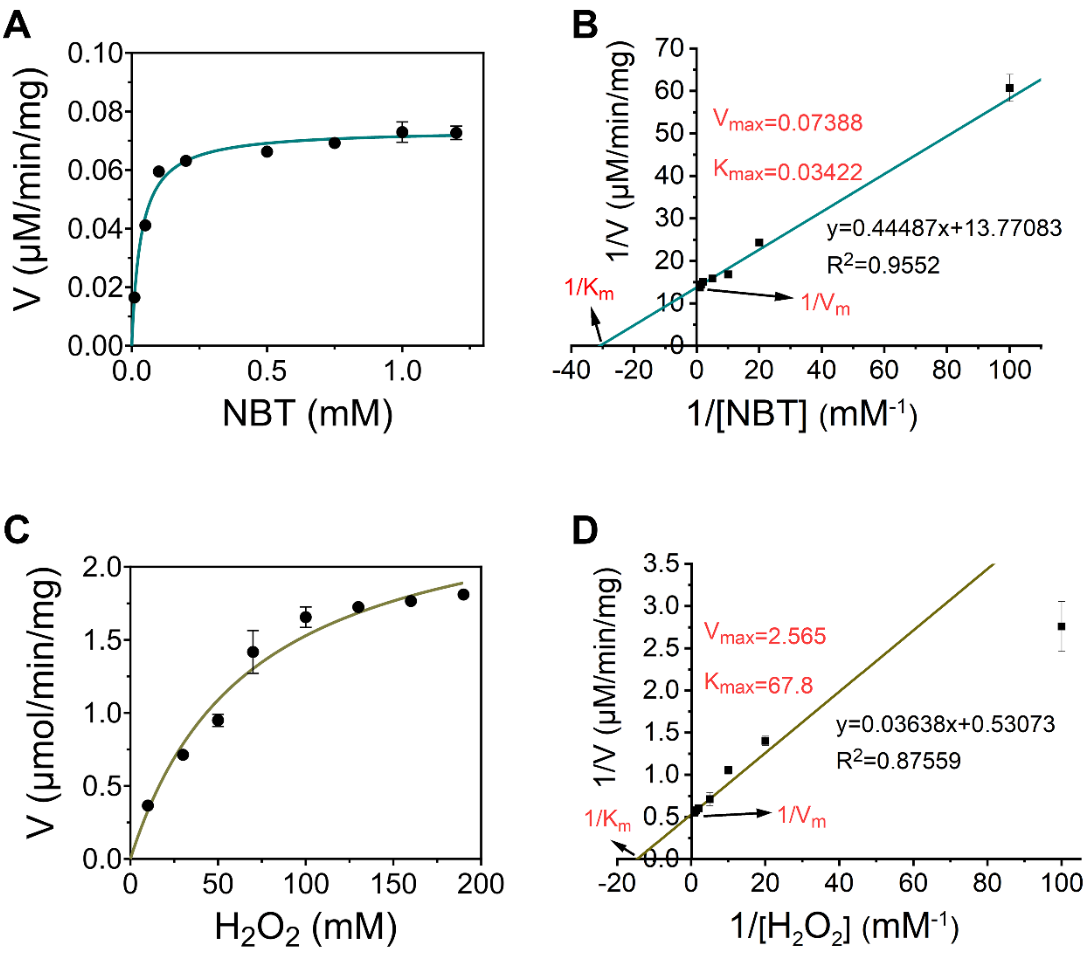

Figure S6. Enzyme kinetics assay. (A, B) Michaelis-Menten plot and Lineweaver-Burk plot of SOD-like Activity for AC-1@MXene, (C, D) Michaelis-Menten plot and Lineweaver-Burk plot of CAT-like Activity for AC-1@MXene.

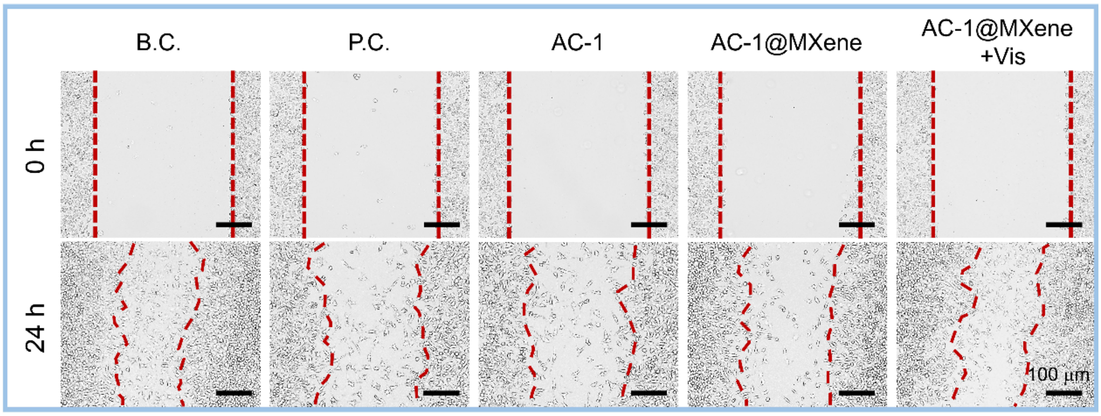

Figure S7. A scratching test was performed to evaluate L929 cell migration ability under oxidative stress. Scale bar: 100 μm.

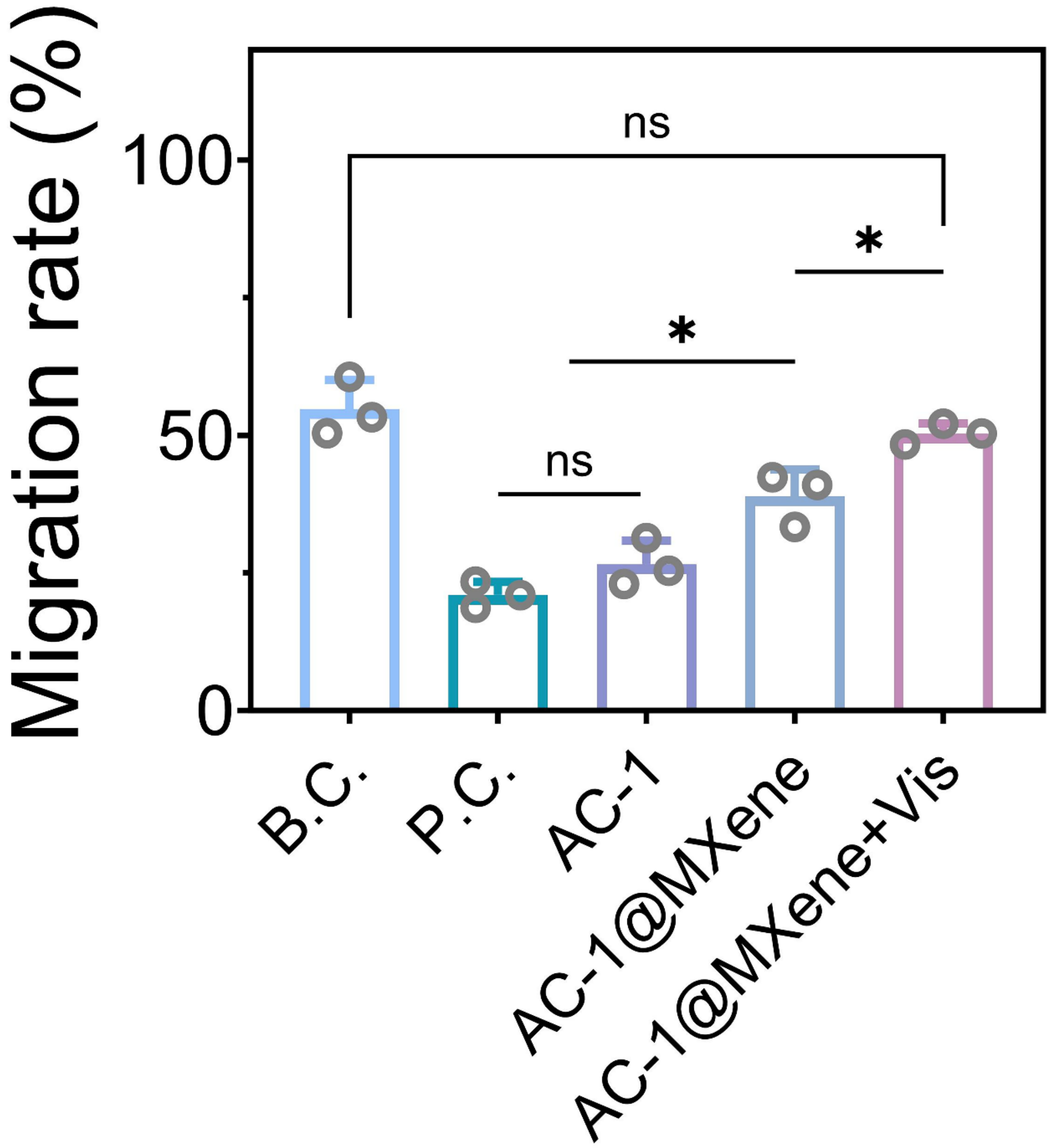

Figure S8. Quantitative results of cell migration rate (n = 3). Values are expressed as the mean ± SD. *$p < 0.05$.

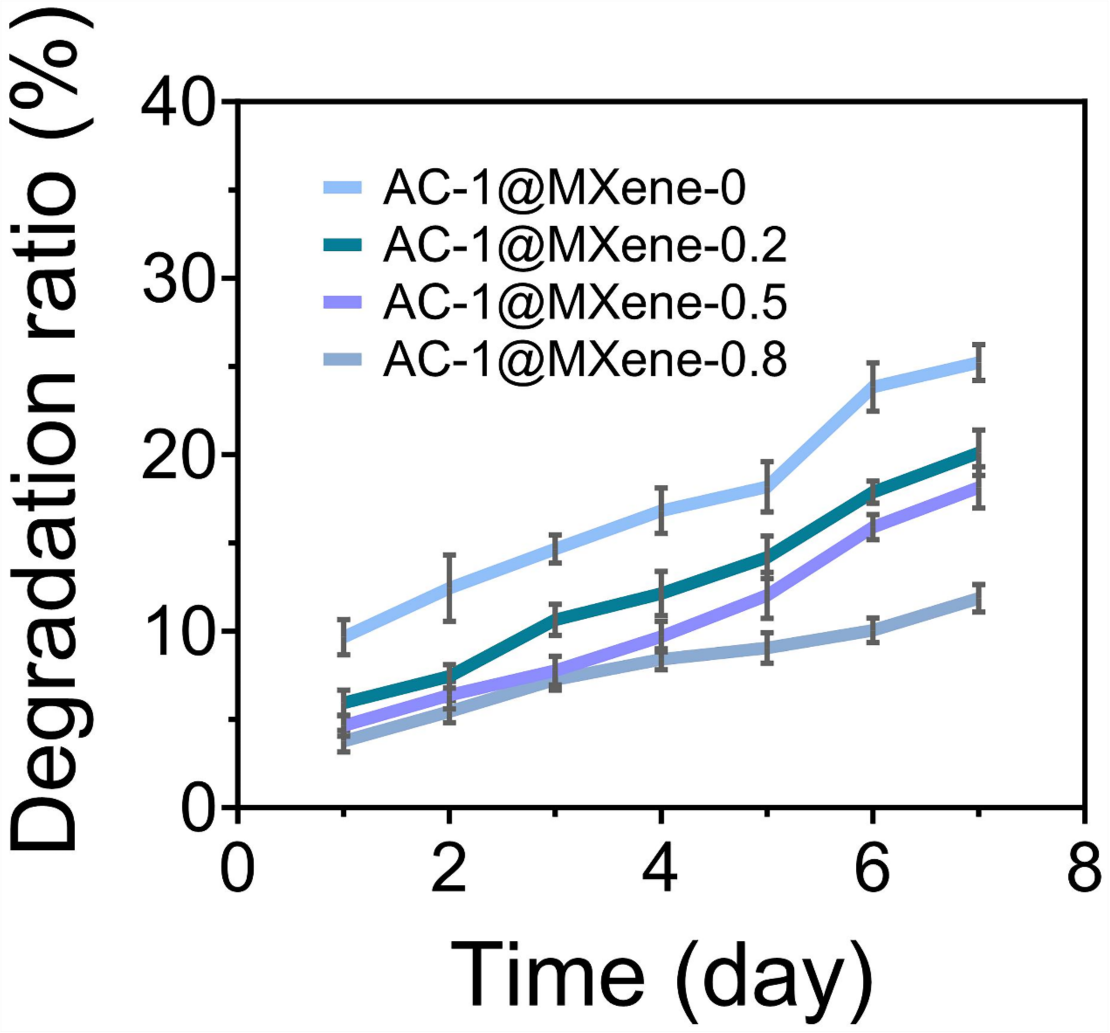

Figure S9. Comparison of degradation lines of hydrogel materials within 7 days.

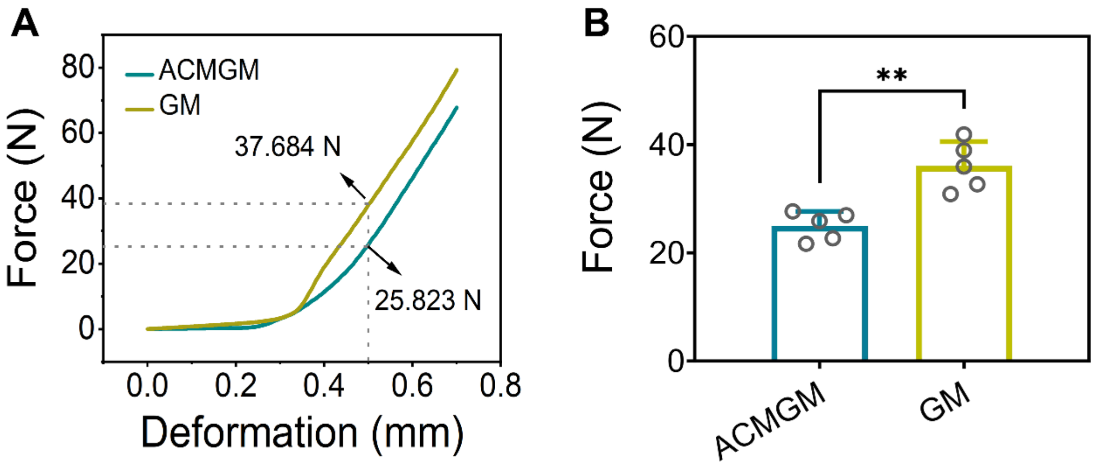

Figure S10. Mechanical testing of microneedles. (A) Deformation-force curves; (B) Quantitative results of force (n = 5). Values are expressed as the mean ± SD. For inter-groups comparison, **P < 0.01.

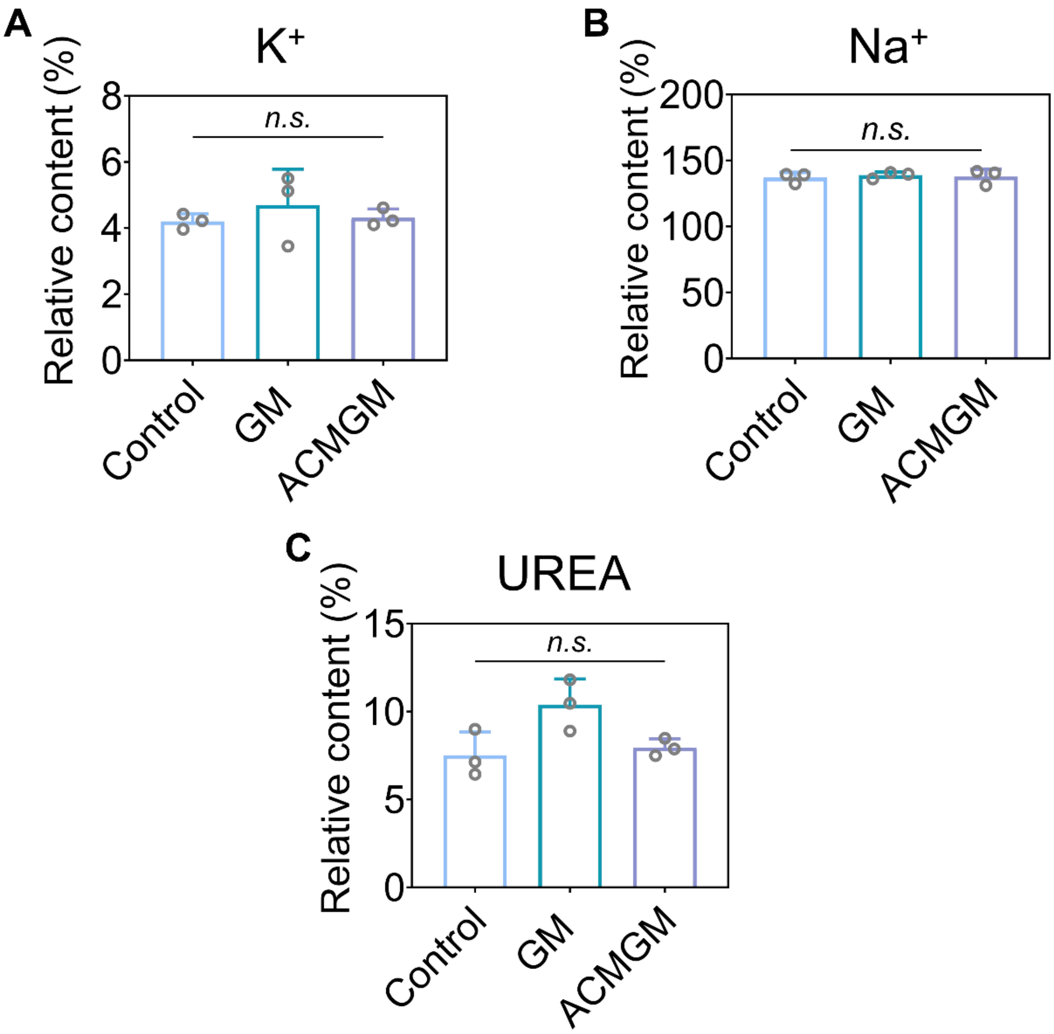

Figure S11. Quantitative results of blood biochemical (A) K^+, (B) Na^+, and (C) UREA index data in SD rats (n = 3). Values are expressed as the mean ± SD. For inter-groups comparison, *n.s.* indicated no significance.

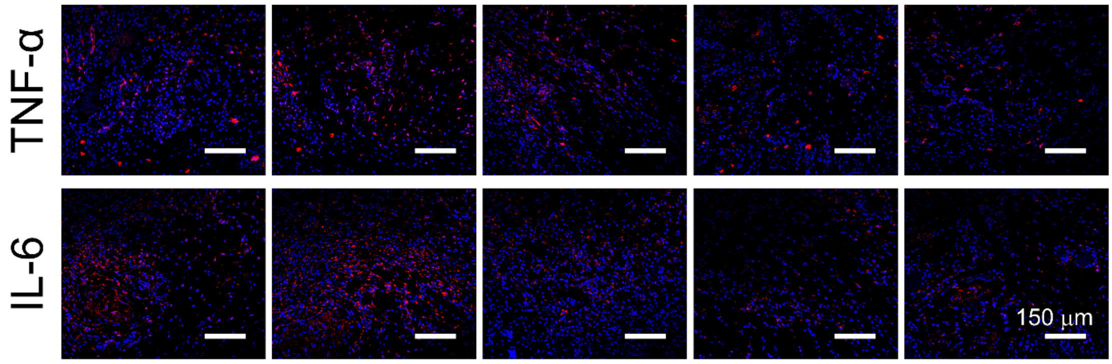

Figure S12. Immunofluorescence staining of TNF-α, IL-6 at day 7. Scale bar: 150 μm.

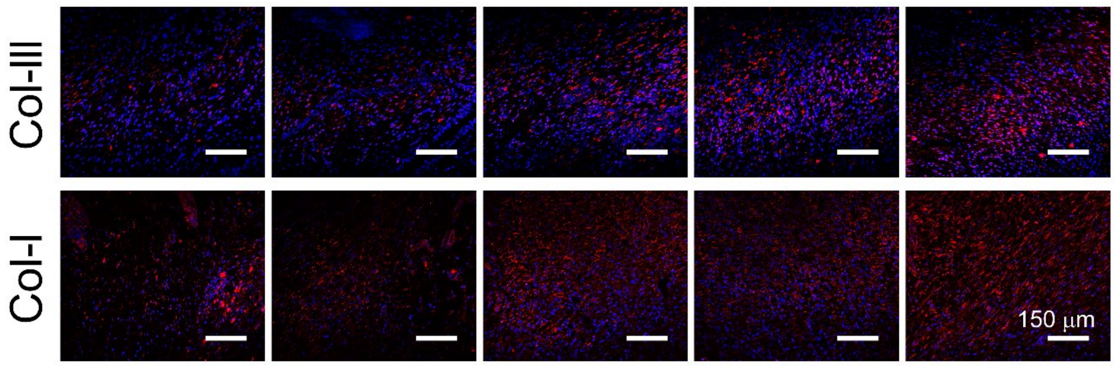

Figure S13. Immunofluorescence staining of Col-lll, Col-l at day 14. Scale bar: 150 μm.

.

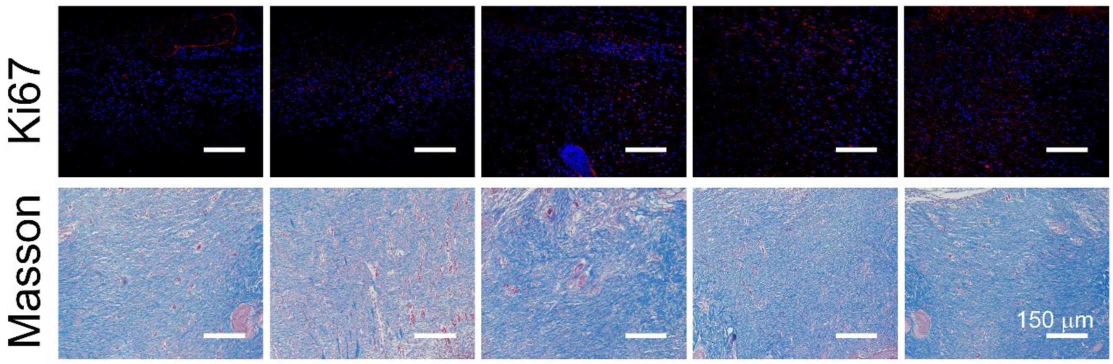

Figure S14. Immunofluorescence staining of Ki67 and Masson at day 14. Scale bar: 150 μm.

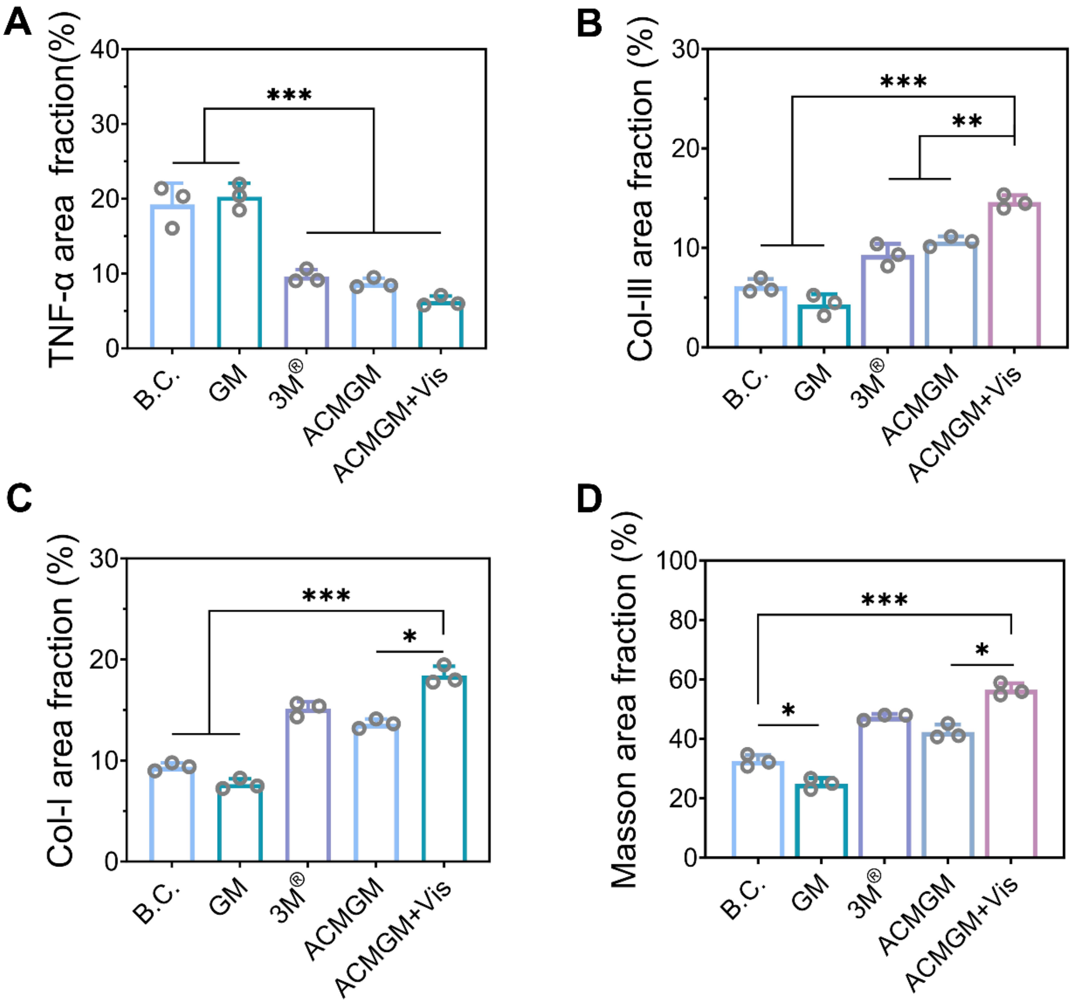

Figure S15. Quantitative results of (A) TNF-α，(B) Col-lll，(C) Col-l and (D) Masson (n = 3). Values are expressed as the mean ± SD. For inter-groups comparison, *p < 0.05, **P < 0.01, ***P < 0.001.

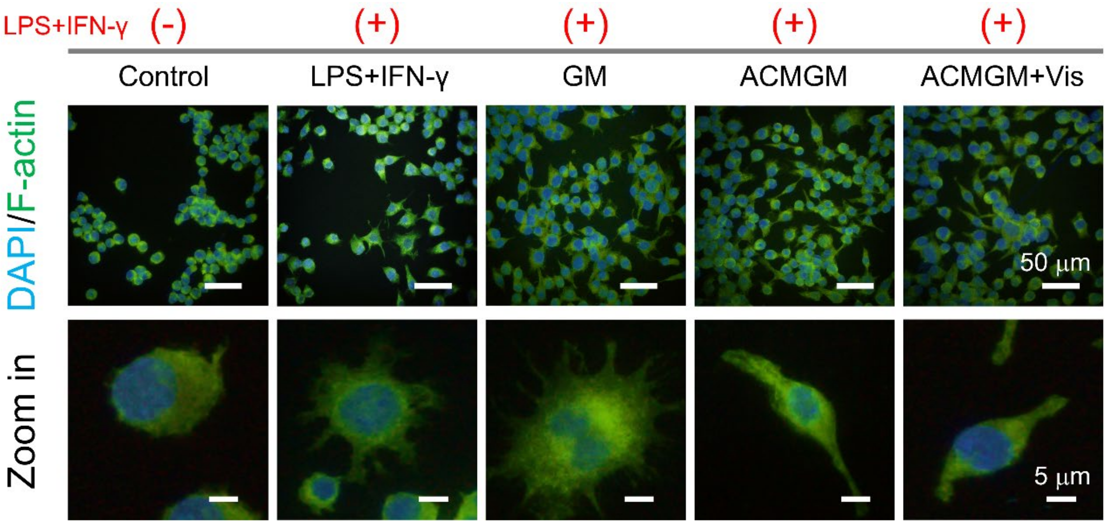

Figure S16. Immunofluorescence staining of the cytoskeleton in RAW 264.7 cells. Scale bar: 50

μm and 5 μm.

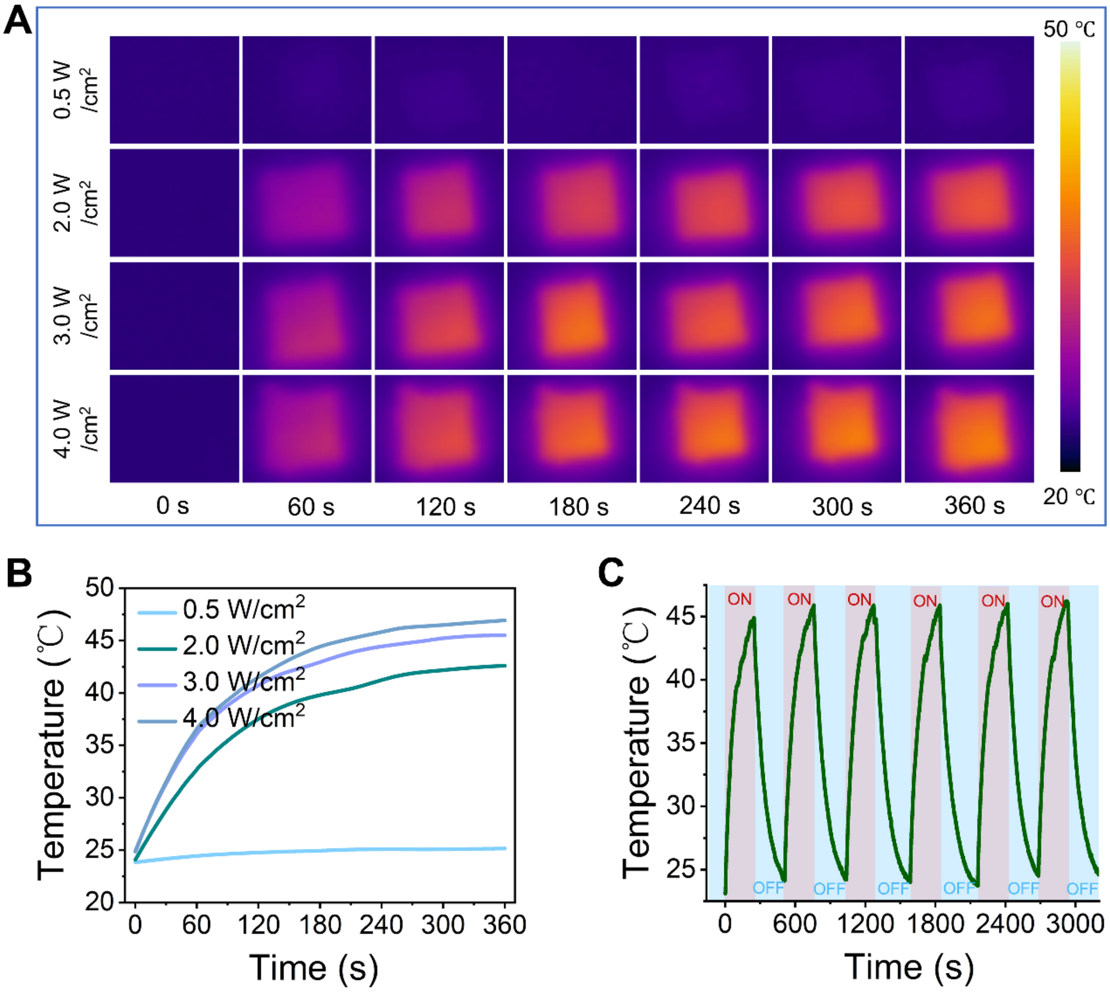

Figure S17. Photothermal testing. (A) Photothermal response graph of AC-1@MXene, (B) Photothermal response curve of AC-1@MXene, (C) Photothermal cycling diagram of AC-1@MXene.
